# Numerical investigation of the effect of rectangular and semicircular cavities filled with phase change materials installed on the solar wall

**DOI:** 10.1007/s11356-023-28244-y

**Published:** 2023-07-12

**Authors:** Jawed Mustafa, Saeed Alqaed, Fahad Awjah Almehmadi, Shahid Husain, Basharat Jamil, Mohsen Sharifpur

**Affiliations:** 1 Mechanical Engineering Department, College of Engineering, Najran University, P.O. Box (1988), Najran, 61441 Saudi Arabia; 2 Department of Applied Mechanical Engineering, College of Applied Engineering, Muzahimiyah Branch, King Saud University, P.O. Box 800, Riyadh, 11421 Saudi Arabia; 3 Department of Mechanical Engineering, Zakir Husain College of Engineering and Technology, Aligarh Muslim University, Aligarh, 202002 India; 4 High Temperature Processes Unit, IMDEA Energy Institute, Av. Ramón de La Sagra, 3, Móstoles, 28935 Madrid, Spain; 5 Department of Mechanical and Aeronautical Engineering, University of Pretoria, Pretoria, South Africa; 6 Department of Medical Research, China Medical University Hospital, China Medical University, Taichung, Taiwan

**Keywords:** Solar energy, Building, Numerical method, Phase change materials

## Abstract

The use of alternative energy sources, particularly solar energy, in buildings is rising and spreading around the globe. In this paper, a solar wall is analyzed using a numerical method. On the wall, a number of obstacles are placed in two shapes, rectangular (REC) and semicircular (SEC). The cavities are filled with organic phase-change materials. This study was performed in 7 h in the absence of solar radiation on the wall for different dimensions of obstacles in 5 different modes. Various temperatures have been investigated, including exhaust air temperature (TAR), Trombe wall temperature (TWL), and mean volume % of molten PCM in cavities. COMSOL software is used to carry out this numerical study. The results of this study showed that the use of SECs compared to RECs causes the TWL to be higher. In the most extreme case, at a 16 cm aspect ratio, the use of SECs gives a 2.1 °C increase in TWL relative to the REC one. The outlet TAR is also increased by the usage of SECs. The use of larger dimensions of the cavities has increased the TAR leaving the wall so that the TAR after 7 h of the absence of solar radiation, in the most significant case of SECs, was more than 295.5 K. The use of SECs also increases the PCM freezing time. In the largest case of cavities, using SECs increases the freezing time by 15 min compared to RECs.

## Introduction

The extreme dependence of industrial societies on energy resources, especially fossil fuels, and their increasing consumption have caused their depletion. Fossil fuels such as oil and coal are finite and take a long time to regenerate. They produce large amounts of various toxic gases each year. Carbon dioxide, which is the most important gas produced by petroleum products, even natural gas, which is the cleanest type of fossil fuel, is a non-toxic gas; however, its greenhouse type is very dangerous that destroys the ozone layer (Ascione, Bianco et al. 2021, Yang, Wang et al. 2021). Burning petroleum products causes harmful gases to release, resulting in the production of acid rain that has destructive effects on living organisms, forests, and even building materials. Clean energies do not have such negative effects on the environment (Mustafa, Alqaed et al. 2021, Xiong, Altnji et al. 2021, Aghakhani and Afrand 2022). By using renewable energy, the environmental problems resulting from the consumption of fossil fuels and the amount of air pollution will be reduced, and saving on the consumption of fossil fuels (non-renewable) will be enhanced (Biwole, Eclache et al. 2013, Al-Abidi, Mat et al. 2014, Barbosa and Ip 2014). Renewable energy is energy that can be returned to nature. Renewable energy sources are stable sources and can be used many times. Renewable energy sources include biomass, water, geothermal, solar, wind, and marine (Karthick, Suresh et al. 2019, Parsa, Yazdani et al. 2022). One of the most important sources of renewable energy is solar, which has attracted the attention of many researchers for a long time. Solar energy is free and can be found worldwide (Aberoumand, Ghamari et al., 2018, Amjad, Jin et al., 2018, Mahdi, Lohrasbi et al., 2019). This kind of energy could be utilized in different ways, e.g. in buildings. Buildings require energy for heating and cooling so that people can live comfortably in them (Hu, He et al. 2017, Sergei, Shen et al. 2020). Using solar energy in buildings can ultimately reduce energy consumption (Izadi and El Haj Assad 2021, Jamil, Ali et al. 2021, Usman, Ali et al. 2021).

Phase change materials (PCMs) can change phase, for instance, from solid to liquid, in an almost constant temperature range. If PCMs are employed in buildings, storing a large amount of solar energy during the day and using the same energy to heat the space during the night is possible. Therefore, some investigators have examined the use of PCMs in buildings. Lin et al. (2005) employed plates containing stable PCM in underfloor heating systems with electric heaters to absorb heat during the night when the price of electricity is low and return heat during the day when the price of electricity is high. They used an enclosure containing the reference and test parts. Zhou et al. )^2015^( evaluated the thermal performance effect of four different geometries of sand and PCM combinations in underfloor heating. It was demonstrated that there are fewer temperature changes by using PCM than by utilizing sand due to the phase change process. The energy release process in using PCM is almost twice as much as using sand in the same experimental conditions. In another study, Aghakhani et al. (2022) revealed the effect of using PCM enclosures with different angles in the walls of a building. They assessed the melting contours and volume fraction of molten PCM by changing the dimensions, angle, and number of PCM enclosures. In the meantime, some researchers have employed PCMs in the thrombus wall in buildings so that they catoergy in the thrombus wall and improve their efficiency. In one of these studies, Tlili and Alharbi (2022) numerically examined the impact of PCM in thrombus walls on the freezing/melting processes, by changing the location of PCM blocks in the wall and conducting a transient study. They evaluated the freezing time of PCM and demonstrated that the lower blocks in the wall lost their temperature earlier and solidified faster in the presence of PCM.

Using solar energy and other new and renewable energy in buildings can significantly reduce energy consumption (Kara and Kurnuç 2012, Saadatian, Sopian et al. 2012, Zalewski, Joulin et al. 2012). There are several ways to use solar energy in buildings. One of these methods is to use Trombe walls in buildings. These walls can be used to move air or heat the house (Bojić, Johannes et al. 2014, Bellos, Tzivanidis et al. 2016, Omrany, Ghaffarianhoseini et al. 2016). The short window of time during which solar energy is available is one of the issues and restrictions with utilizing it. Utilizing PCM in the wall is one strategy for storing solar energy (Jamil, Ali et al. 2021, Lawag and Ali 2022). PCMs have the capacity to store thermal energy in their bodies, such as solar thermal energy. (Elghamry and Hassan 2020, Khetib, Alotaibi et al. 2021, Oluah, Akinlabi et al. 2021). With this in mind, this paper simulates a Trombe wall with PCM. On the wall, there are a number of obstacles with two shapes of semicircle and rectangle full of PCM. The varying dimensions of obstructions were studied without the benefit of sun hours. Additionally, the impact of two different types of impediments working together on the wall’s efficiency time has been studied.

## Problem definition

**Fig. 1:**
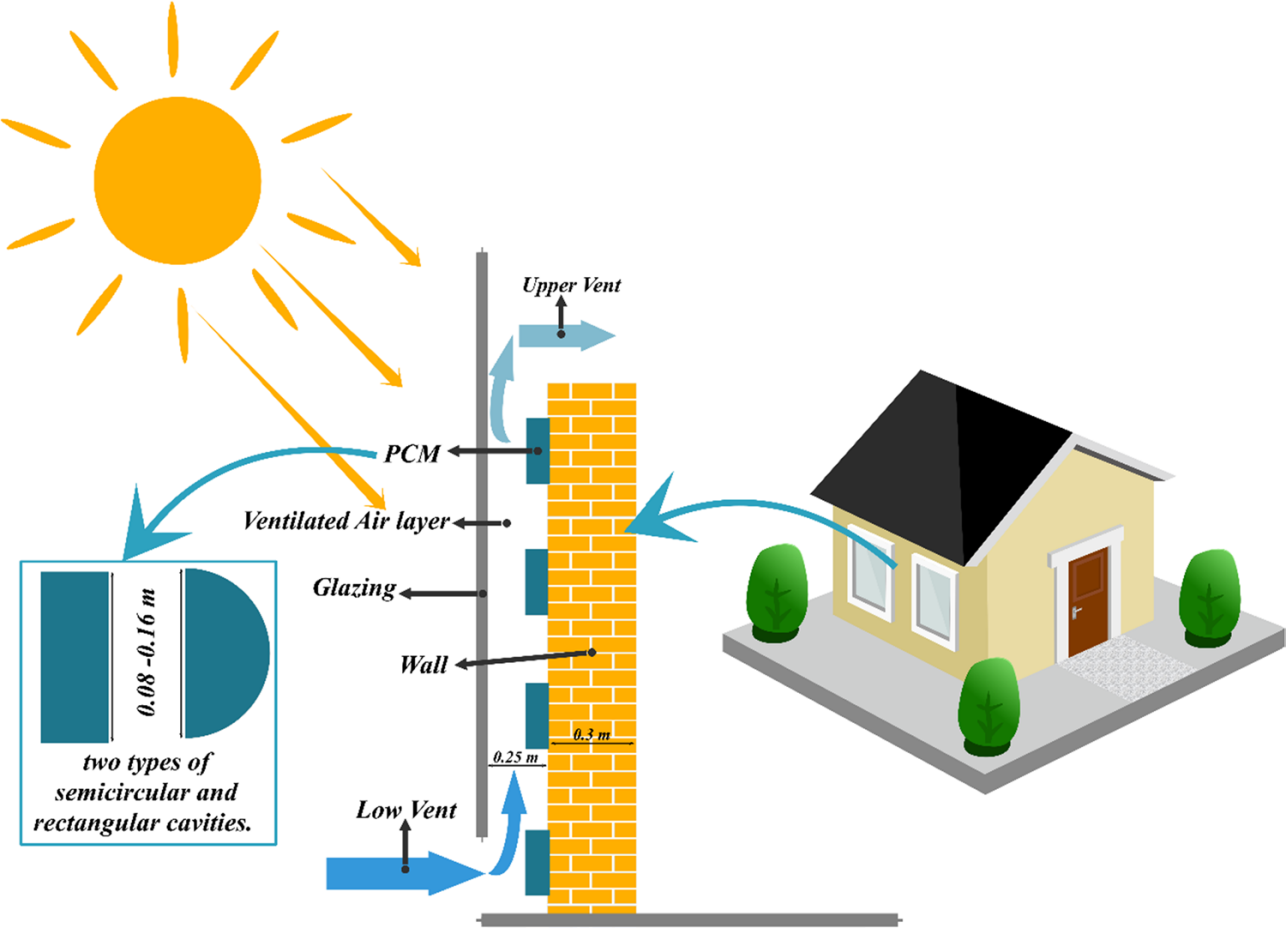
Schematic of the studied wall with two types of SECs and RECs

The wall being analyzed is set up in a space that measures 2 by 3 m and has a ceiling height of 2.8 m. The whole wall is made of concrete, and four cavities with organic PCM have been placed. The two barrier shapes under consideration include a semicircle and a rectangle as shown in Fig. 1. Parameter D (aspect ratio) has been changed from 8 to 16 cm for two obstacles. This parameter represents the radius of the semicircle for the SEC and the width of the rectangle for the REC. This analysis was done for the duration of the night, so the amount of solar radiation was equal to 0, and the temperature of the wall was equal to the temperature of the PCM in the molten state. Also, the inlet air temperature is 10 °C, and the wind speed is considered to be a constant value of 1 m/s. It is noteworthy that the length of the rectangle is chosen in each step so that the volume of PCM used in both REC and SECs is equal. The PCM (gallium) is utilized in the walls (Yang, Tan et al. 2016). Table 1 shows the thermo-physical properties of gallium.

**Table 1:** Thermo-physical properties of gallium

Density (kg/m^3^)	Specific heat (kJ/kg K)	Viscosity (kg/m/s ˟10^−3^)	Thermal conductivity (W/m.k)	Melting point (K)
6094.7	397.6	1.75	33.6767	302.93

Because of the free convection of air at home, buoyancy is also a factor in the equation. For this problem, the Boussinesq approximation has been utilized. The ensuing equations are shown as follows.

1 $$\frac{\partial u}{\partial x}+\frac{\partial v}{\partial y}=0$$

2 $$\frac{\partial u}{\partial t}+\mathrm{u}\frac{{\partial u}}{{\partial x}}+\mathrm{v}\frac{{\partial u}}{{\partial y}}=-\frac{1}{\uprho}\frac{{\partial p}}{{\partial x}}+\frac{1}{\uprho}\left(\frac{\partial }{{\partial x}}\left(\upmu \frac{{\partial u}}{{\partial x}}\right)+\frac{\partial }{{\partial y}}\left(\upmu \frac{{\partial u}}{{\partial y}}\right)\right)$$

3 $$\frac{\partial v}{\partial t}+\mathrm{u}\frac{{\partial v}}{{\partial x}}+\mathrm{v}\frac{{\partial v}}{{\partial y}}=-\frac{1}{\uprho}\frac{{\partial p}}{{\partial y}}+\frac{1}{\uprho}\left(\frac{\partial }{{\partial x}}\left(\upmu \frac{{\partial v}}{{\partial x}}\right)+\frac{\partial }{{\partial y}}\left(\upmu \frac{{\partial v}}{{\partial y}}\right)\right)+\mathrm{g}\upbeta \left(\mathrm{T}-{\mathrm{T}}_{\mathrm{c}}\right)$$

4 $$\frac{\partial T}{\partial t}+\left(\mathrm{u}\frac{{\partial T}}{{\partial x}}+\mathrm{v}\frac{{\partial T}}{{\partial y}}\ \right)=\frac{{\mathrm{k}}_{\mathrm{nf}}}{\uprho {\mathrm{C}}_{\mathrm{P}}}\left(\frac{\partial^2\mathrm{T}}{{{\partial x}}^2}+\frac{\partial^2\mathrm{T}}{{{\partial y}}^2}\right)$$

To solve the PCM solidification and melting issues, it is necessary to solve the equations of mass, momentum, and energy survival in it. The following equations are given for PCM.

5 $$\frac{\partial \rho }{\partial t}+\nabla \cdot \left(\rho \overrightarrow{v}\right)=0$$

6 $$\frac{\partial }{\partial t}\left(\rho \overrightarrow{v}\right)+\nabla \cdot \left(\rho \overrightarrow{v}\overrightarrow{v}\right)=-\overrightarrow{\nabla}p+\rho \overrightarrow{g}+\nabla \cdot \overline{\overline{\tau}}+\overrightarrow{F}$$

7 $$\frac{\partial \left(\rho h\right)}{\partial t}+\nabla \cdot \left(\rho \overrightarrow{v}\ H\right)=\nabla \cdot \left(k\nabla h\right)+S$$

In the momentum equation, term F is defined a ${A}_m\frac{{\left(1-\beta \right)}^2}{\beta^3+0.001}$ according to the references. *A*_*m*_ value is 10^5^. The difference between the melting’s start and finish points leads to the establishment of a parameter known as beta (β). *β* is the paraffin liquefaction fraction, which is 0 before melting begins and one after the paraffin is entirely liquefied and is a value between zero and one in the melting range. *B* is the Boltzmann coefficient with a value of 1.38 × 10^−23^*J*/*K* . The (Arasu and Mujumdar 2012) following equations are given for obtaining *β*.

8 $${\beta}_k=\kern0.5em \left\{\begin{array}{c}\beta =O\kern4.75em if\kern4.25em T<{T}_{solidus}\\ {}\kern2em \\ {}\beta =1\kern4.75em if\kern1.75em T>{T}_{Liquidus}\\ {}\beta =\frac{T-{T}_{solidus}}{T_{Liquidus}-{T}_{solidus}}\kern4.25em if\kern3em {T}_{Liquidus}\le T\le {T}_{solidus}\end{array}\right.$$

## Numerical method and grid study

**Fig. 2:**
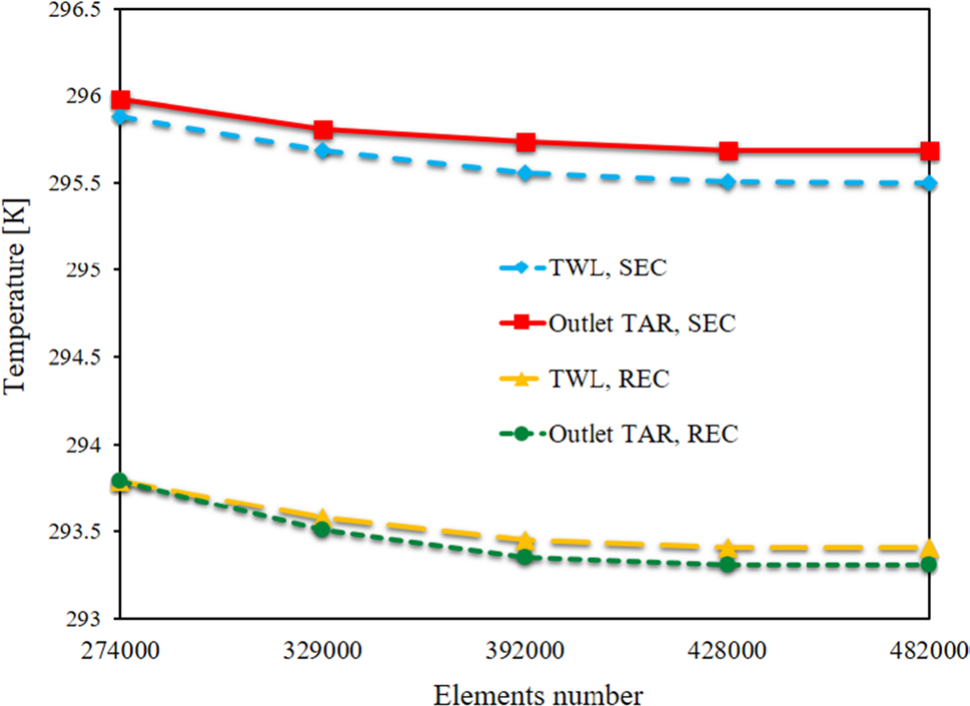
The effect of geometric meshing quality on TWL and outlet TAR in 16 cm aspect ratio for SEC and RECs

COMSOL software was utilized to create the wall simulation. The equations were also algebraized using the finite element approach. A variety of distinct components is investigated in the grid created on the geometry. Finally, the primary mesh for the SEC was chosen at 428 thousand elements, and the number of meshes for the REC was chosen at 465 thousand elements. Figure 2 shows the impact of the number of grid elements on the TAR escaping the wall and the wall’s temperature for the two SEC and RECs.

### Validation

**Fig. 3:**
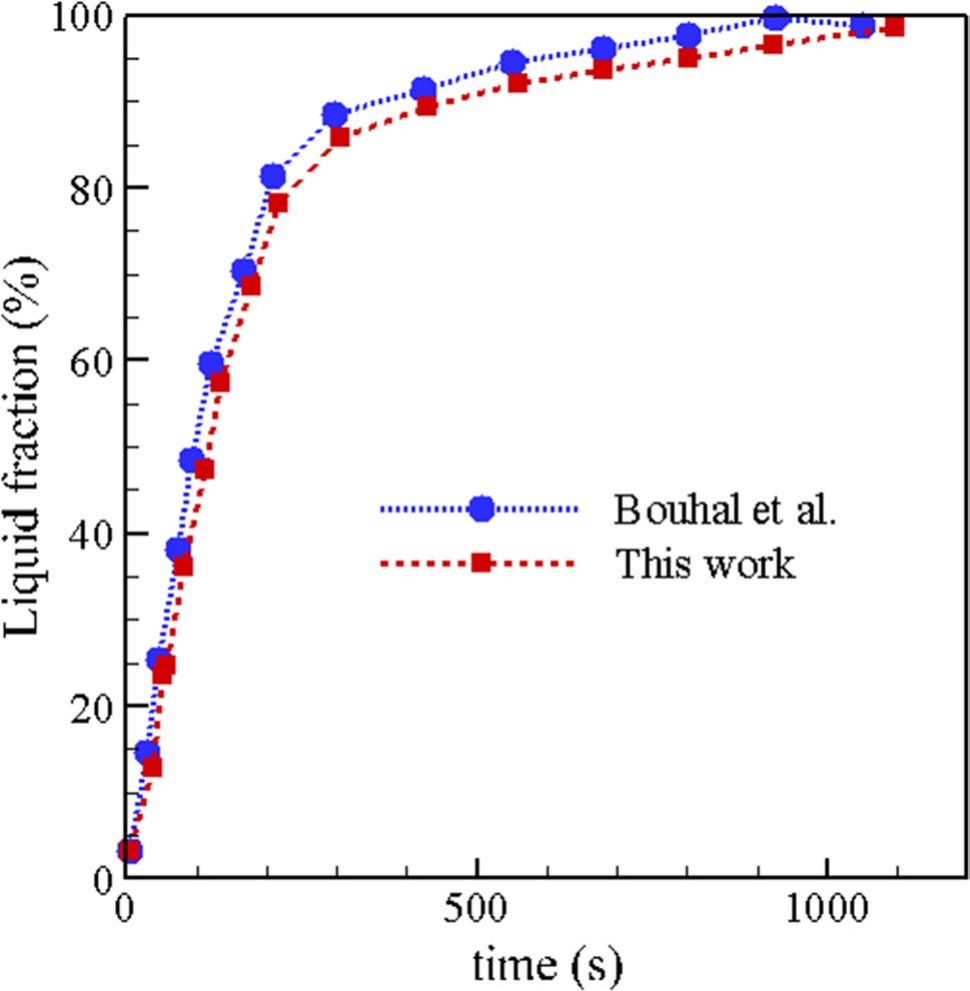
Comparison of melt PCM between the present work and Bouhal et al. (2018)

To verify the results, a validation using the paper Bouhal et al. (2018) has taken place. In this validation, which is given in Fig. 3, the amount of molten PCM at different times between the present work and the article (Bouhal, ed-Dîn Fertahi et al. 2018) is given. The findings of this simulation are acceptable given the error of less than 5% between this study and the validation data.

## Results and discussion

**Fig. 4:**
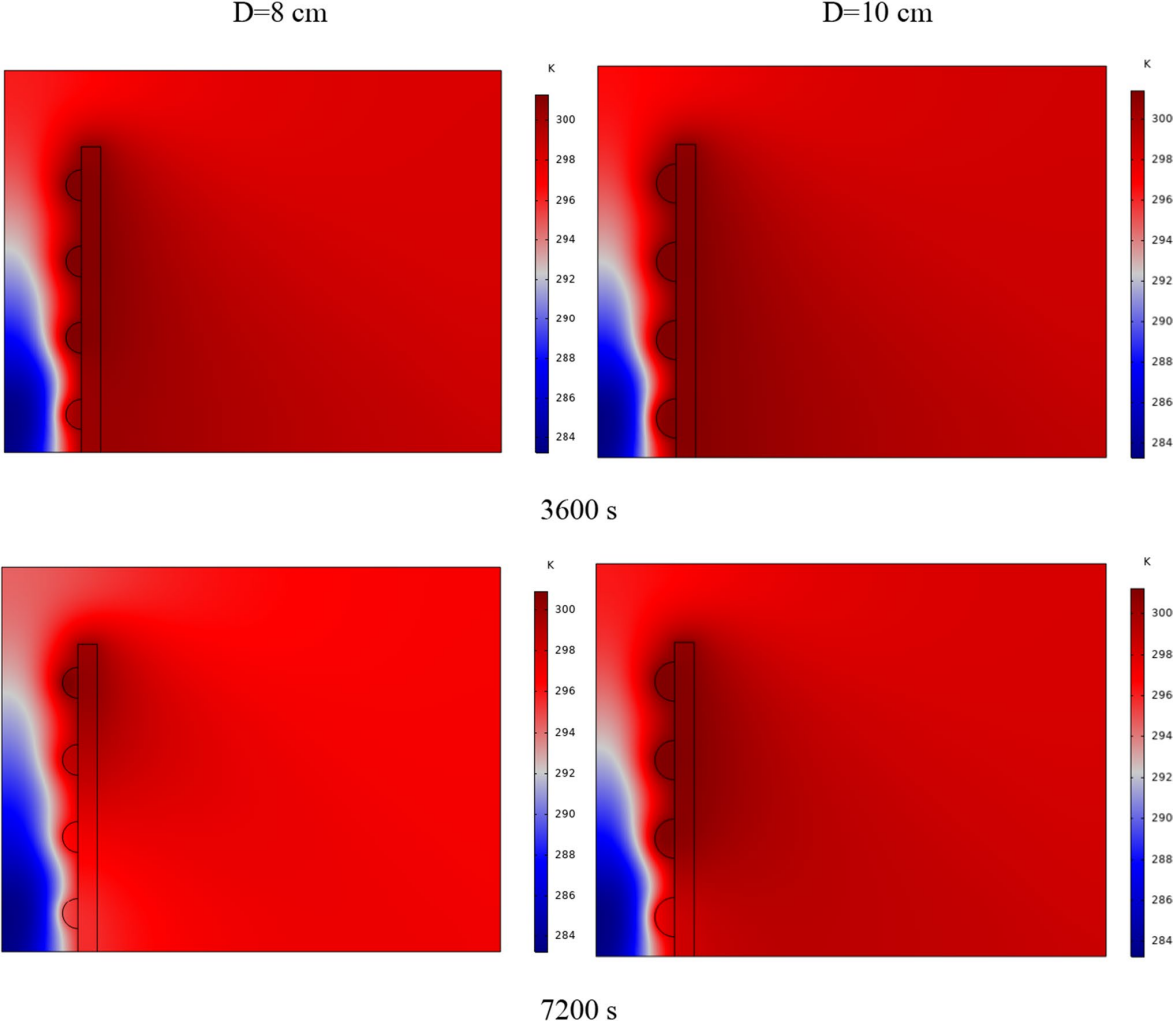

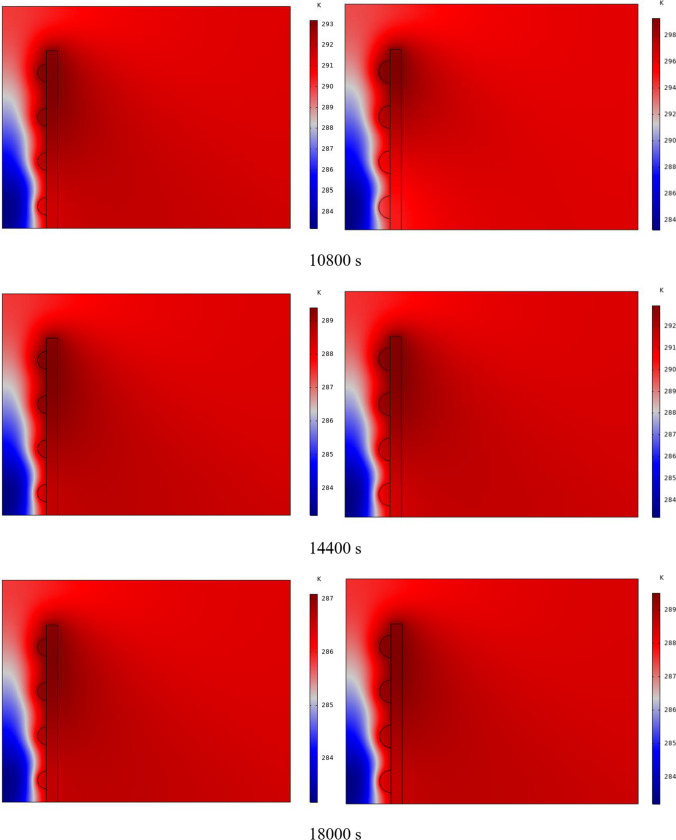

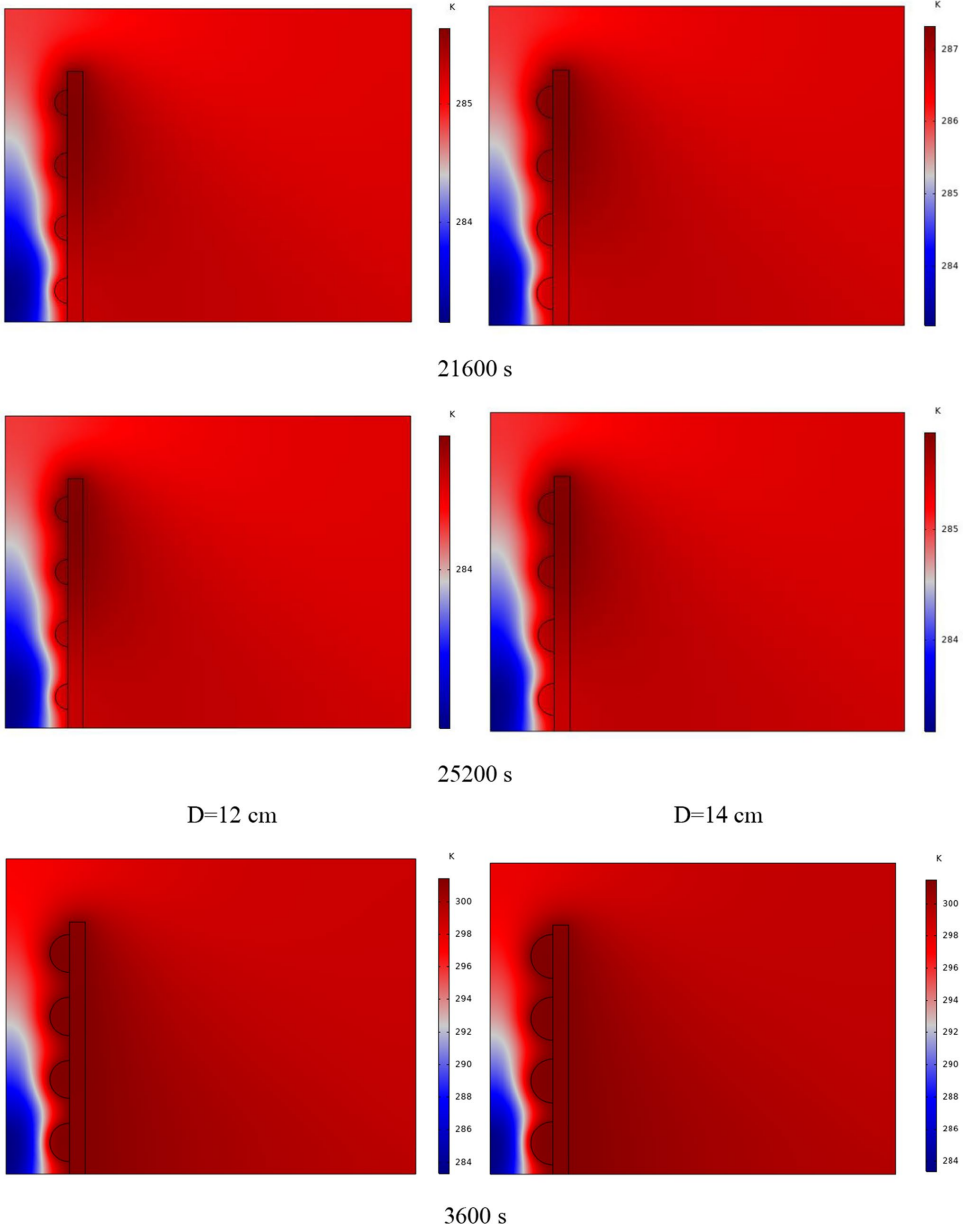

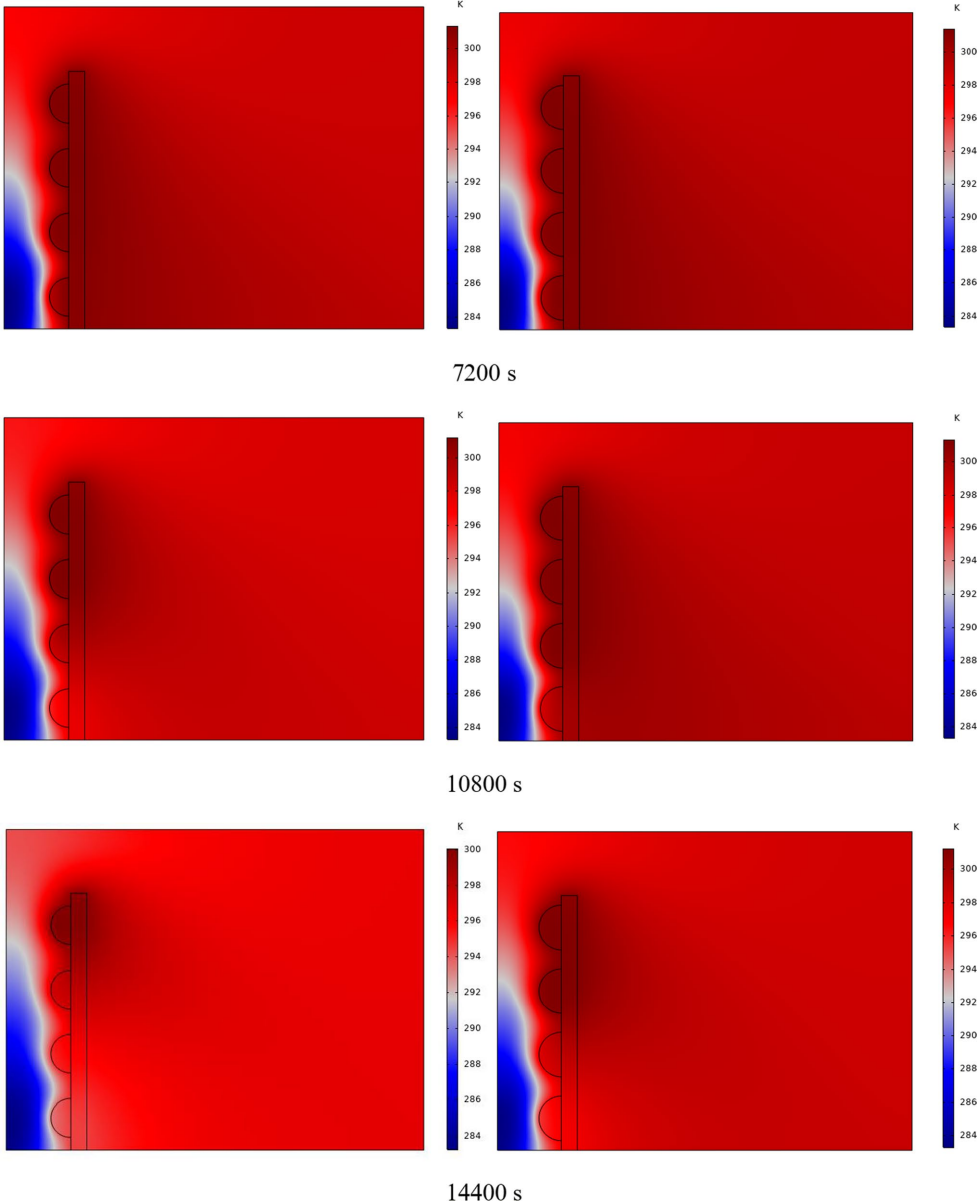

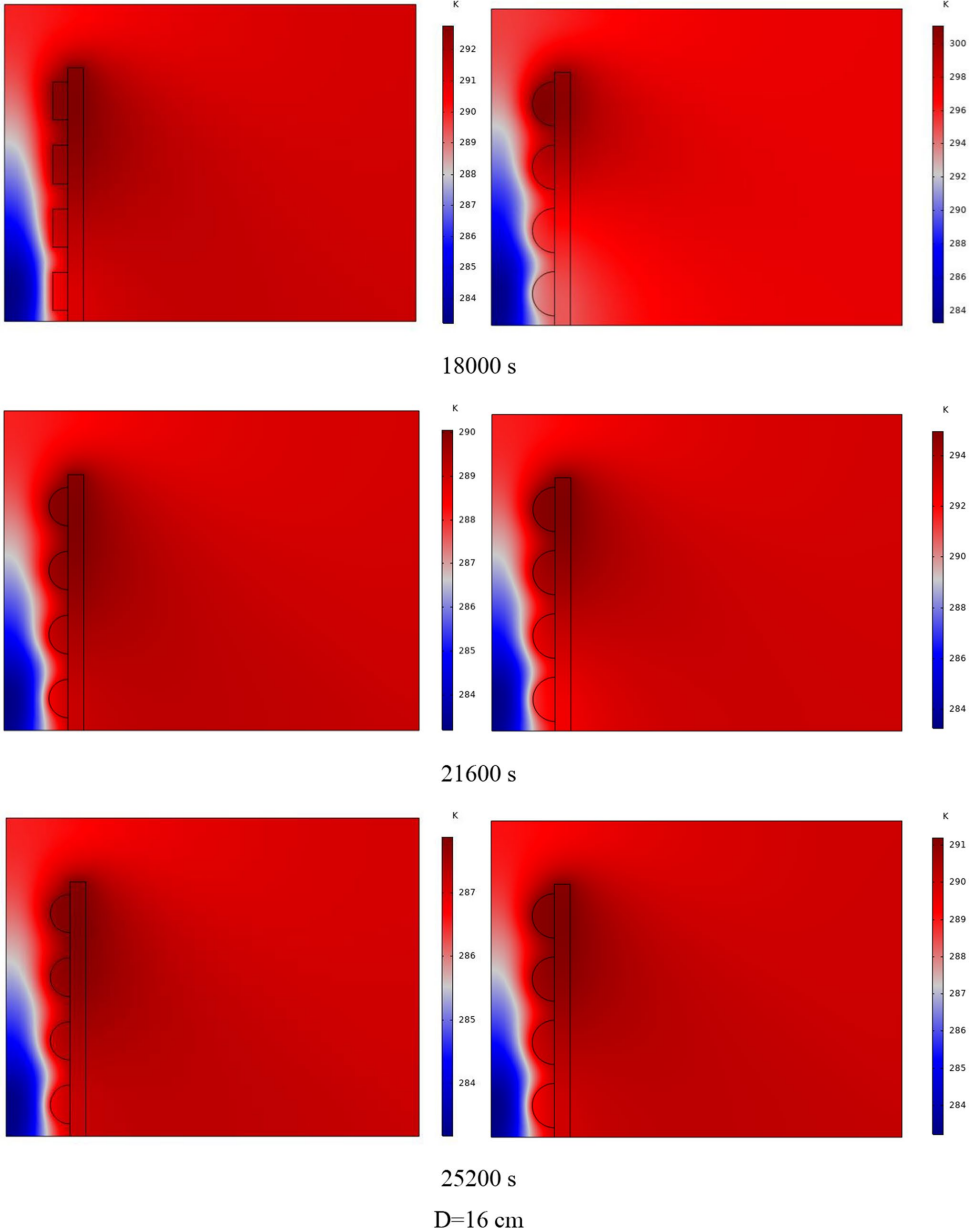

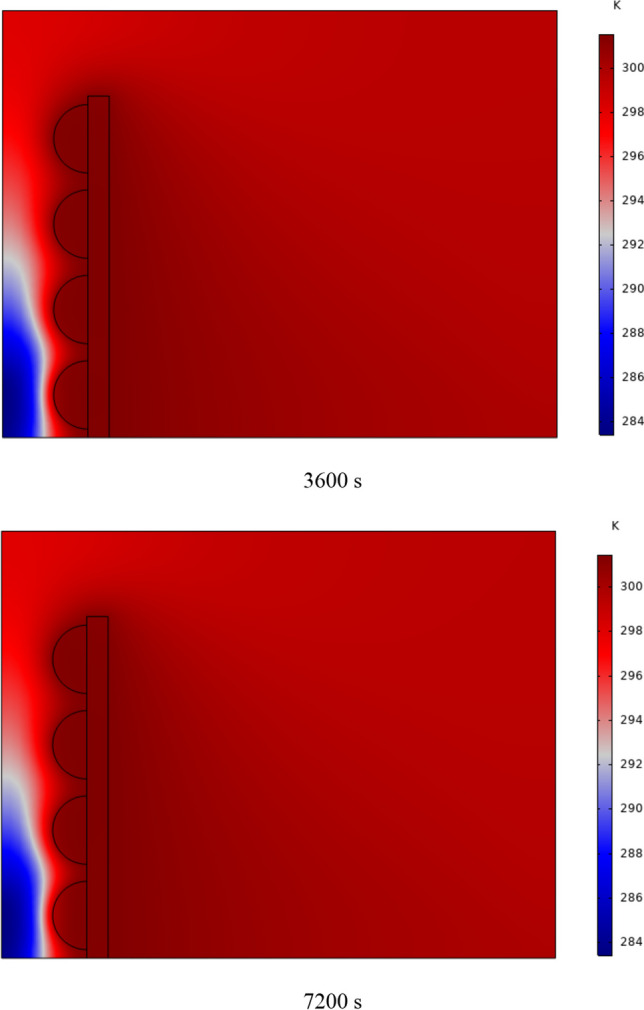

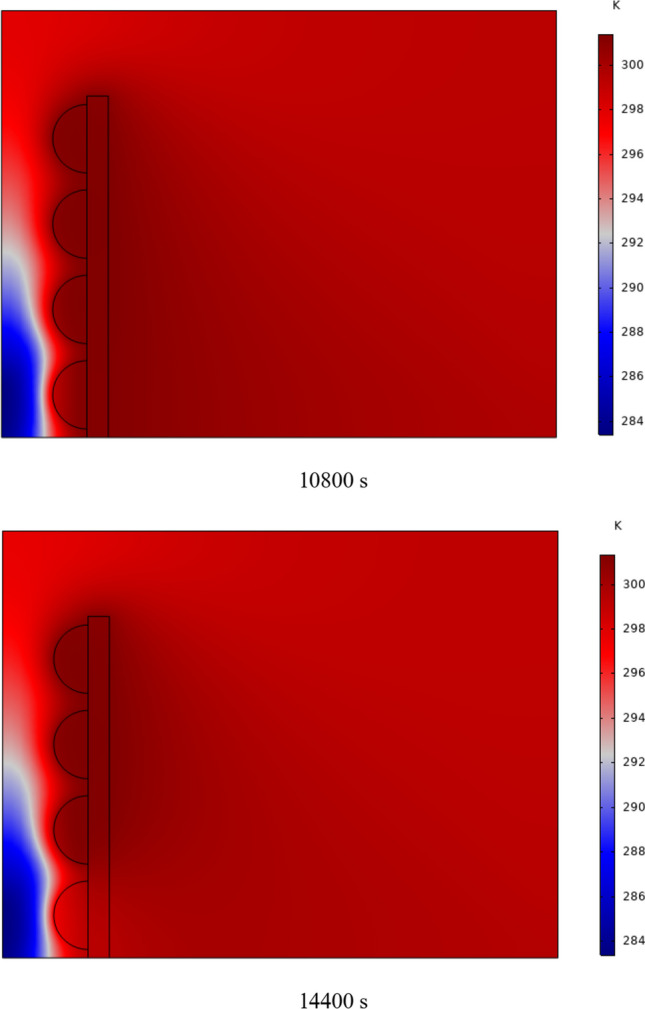

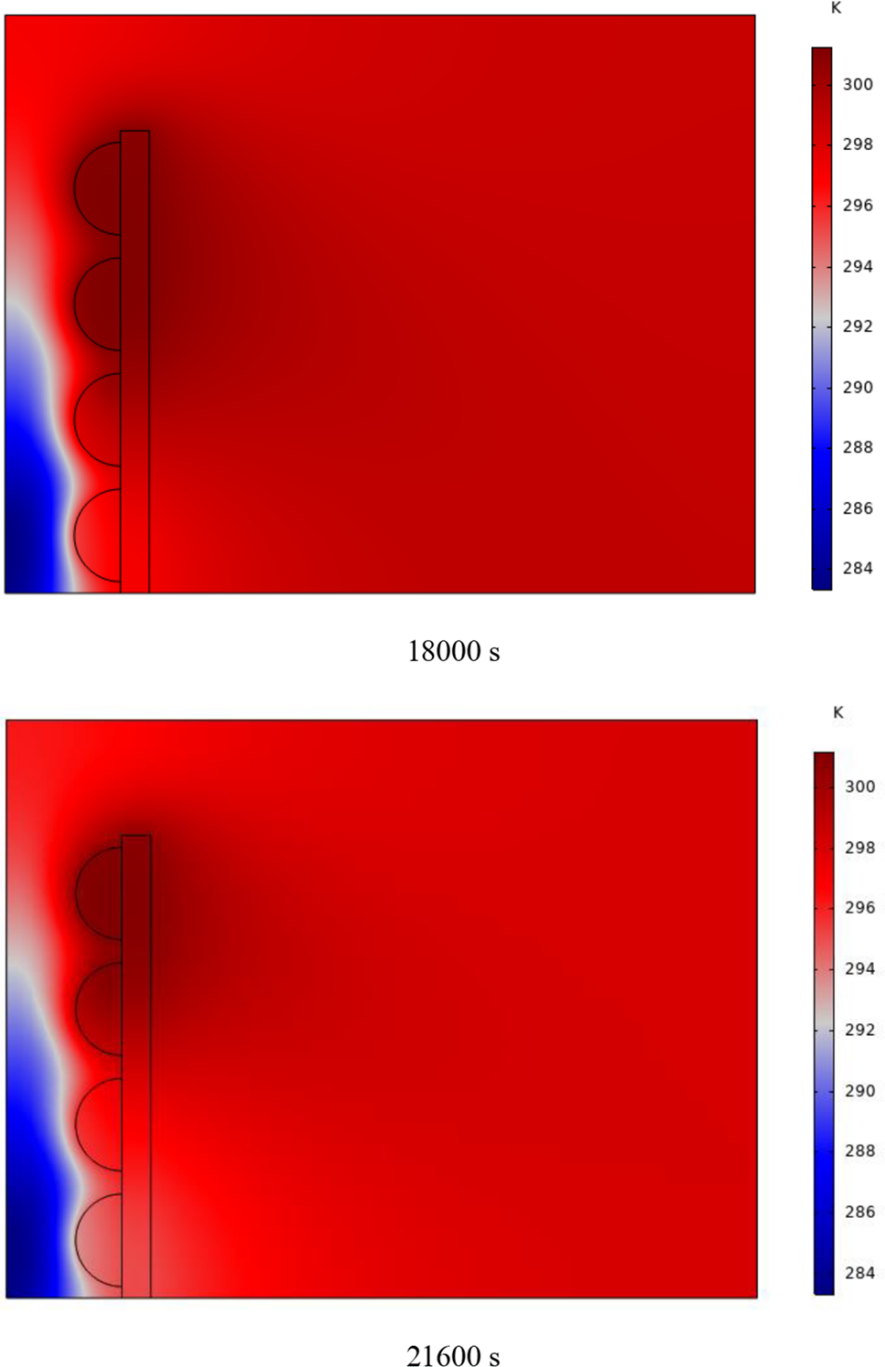

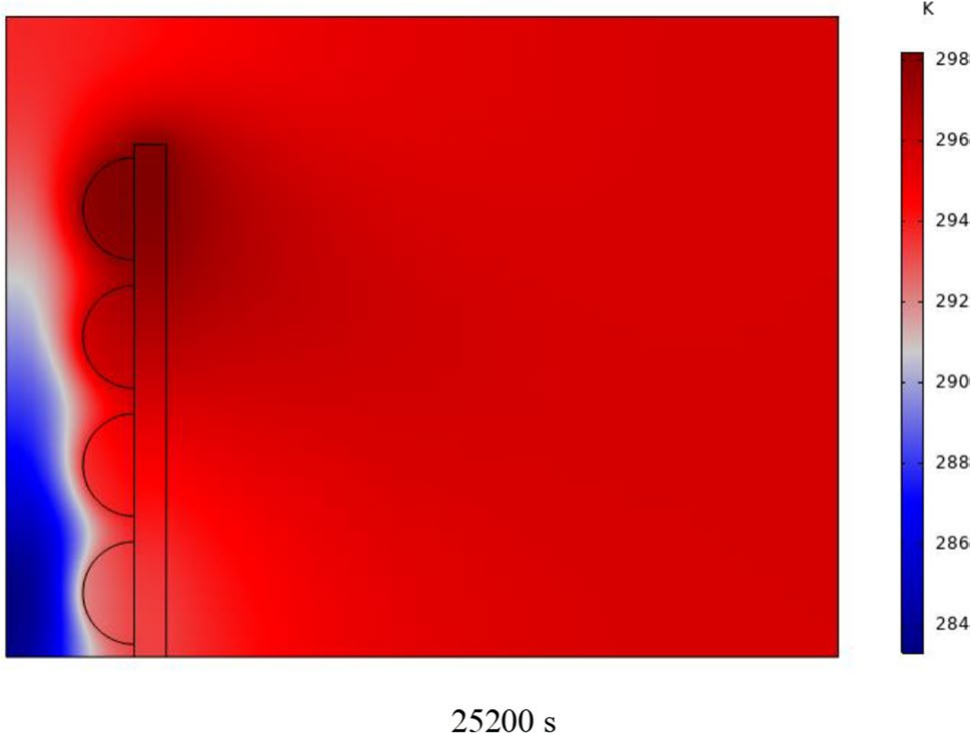
Temperature meter on the wall from 1 to 7 h for (D) 8 to 16 cm for SECs

In Fig. 4, the temperature contour on the wall is enclosed for 1 to 7 h for (D) 8 to 16 cm for SECs. According to the wall, it can be seen that the amount of TWL is largely dependent on the obstacles. In the absence of solar radiation, the wall, which has a low heat capacity, loses its temperature in a short time. These cavities, despite the PCM, maintain the TWL due to the latent heat inside. So, the parts of the wall where the PCM solidifies faster in front of the cavities lose their temperature sooner. The hottest part of the wall is the top, where the molten PCM remains most of the time. The larger the barrier, the more PCM is used in front of the wall, so the molten PCM stays in front of the barrier for longer. So, for more time, the TWL has been in high value. At an aspect ratio of 8 cm, the TWL decreased slightly in time, and even at 7 h, the TWL approached the inlet TAR.

**Fig. 5:**
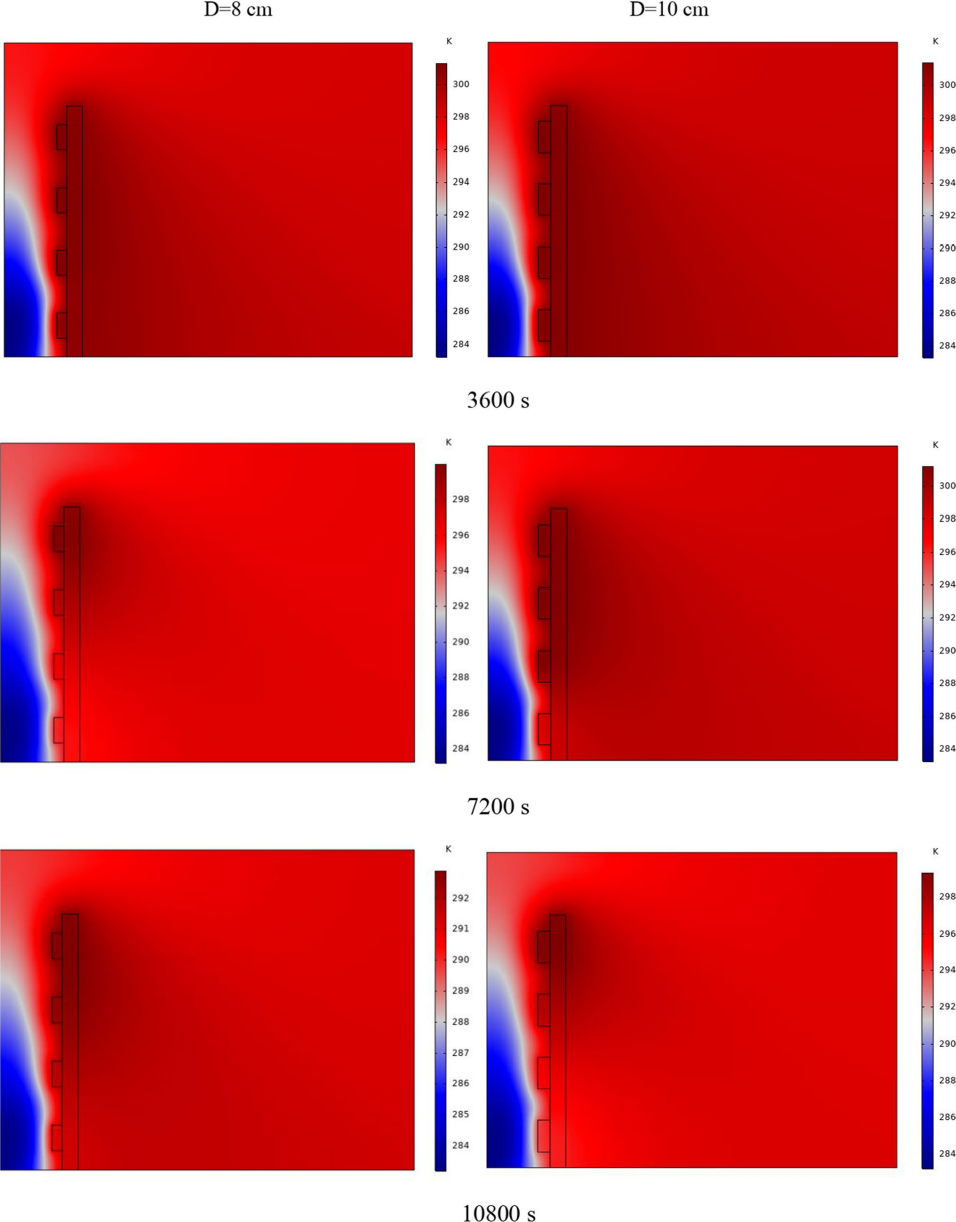

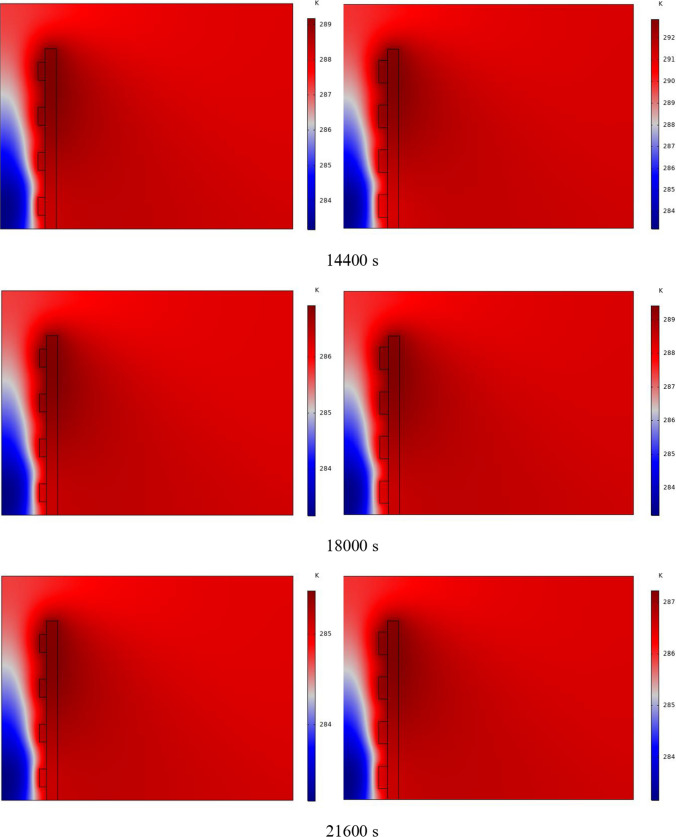

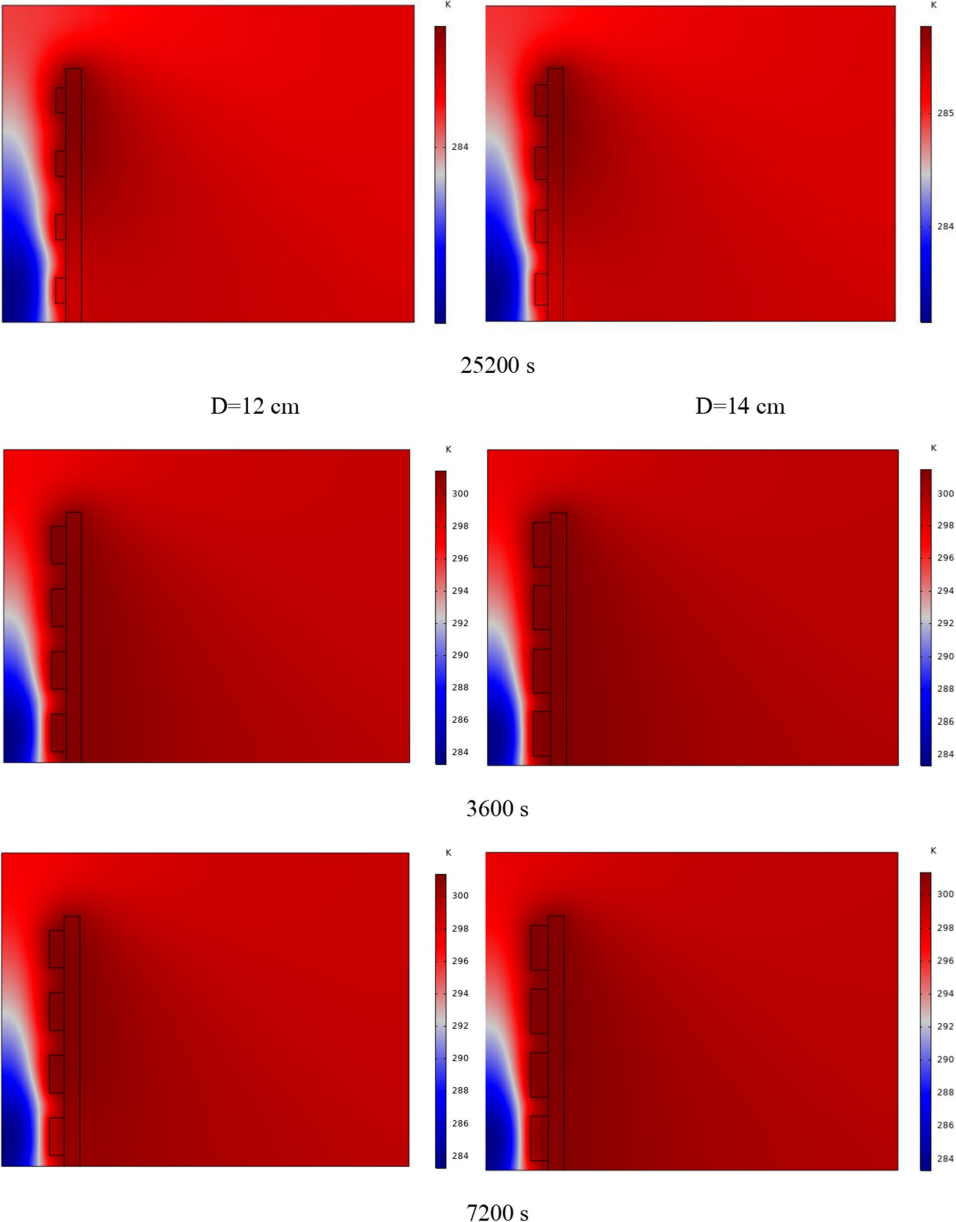

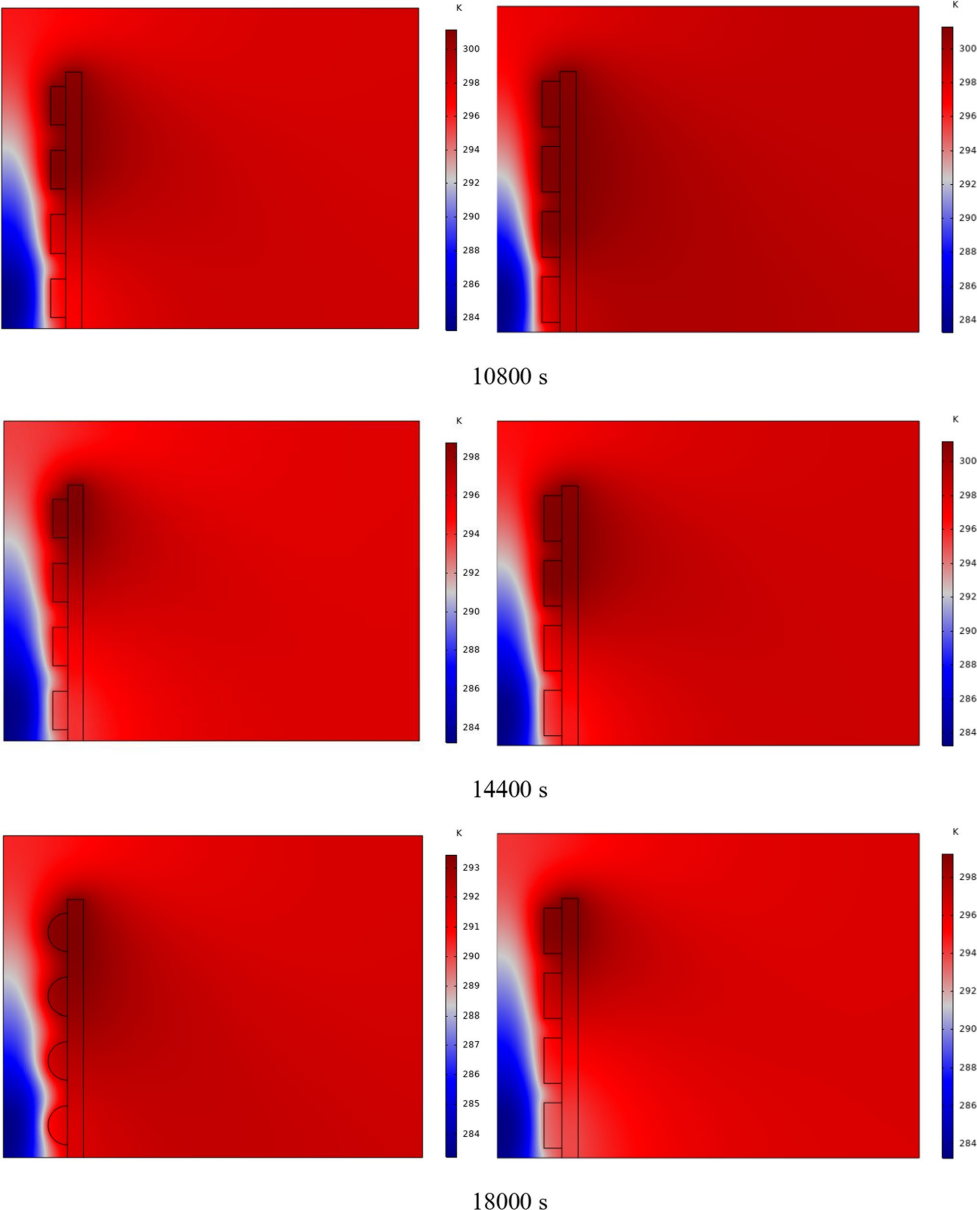

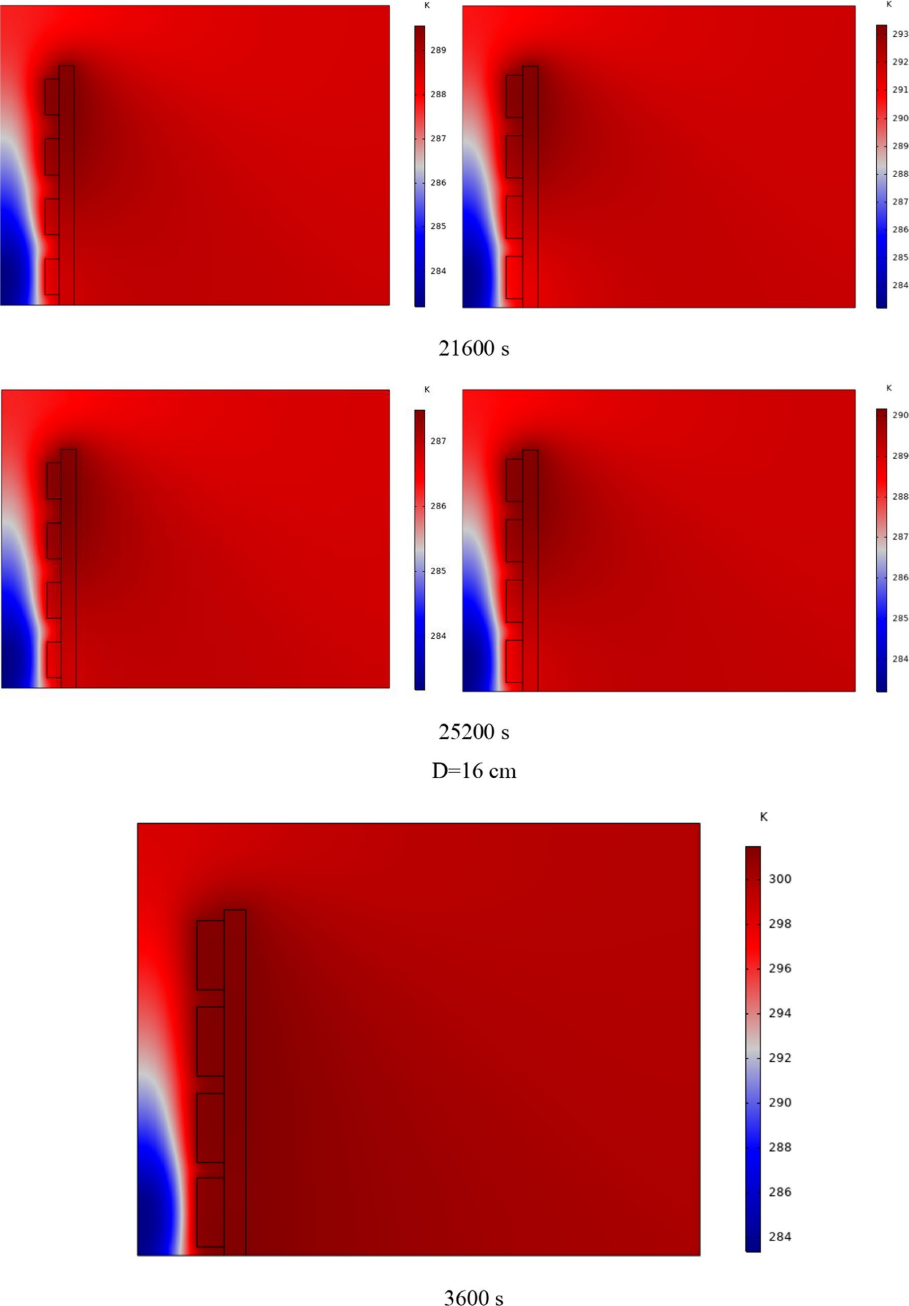

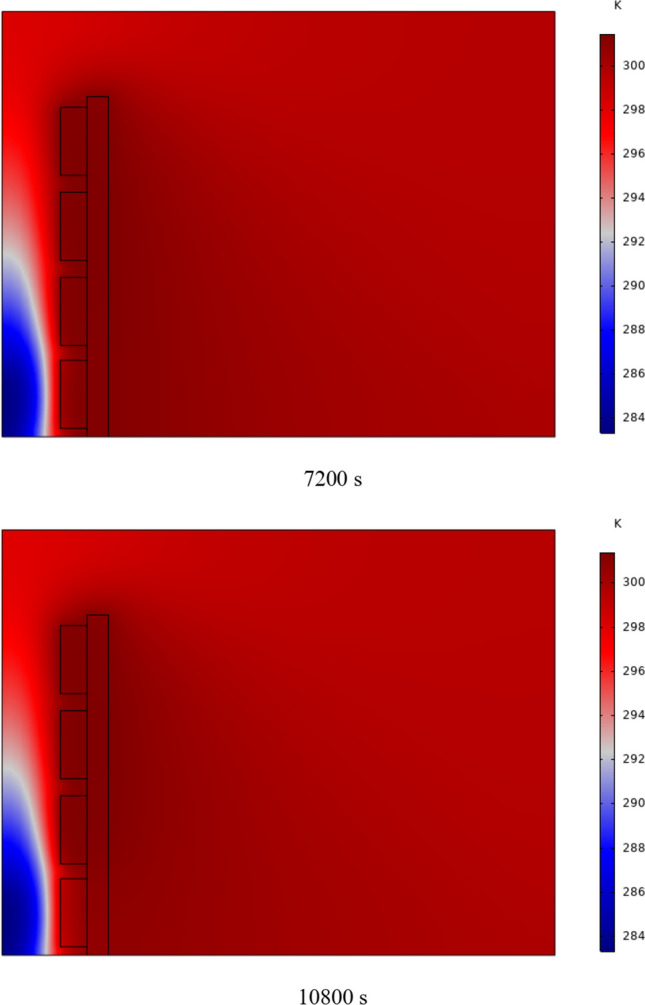

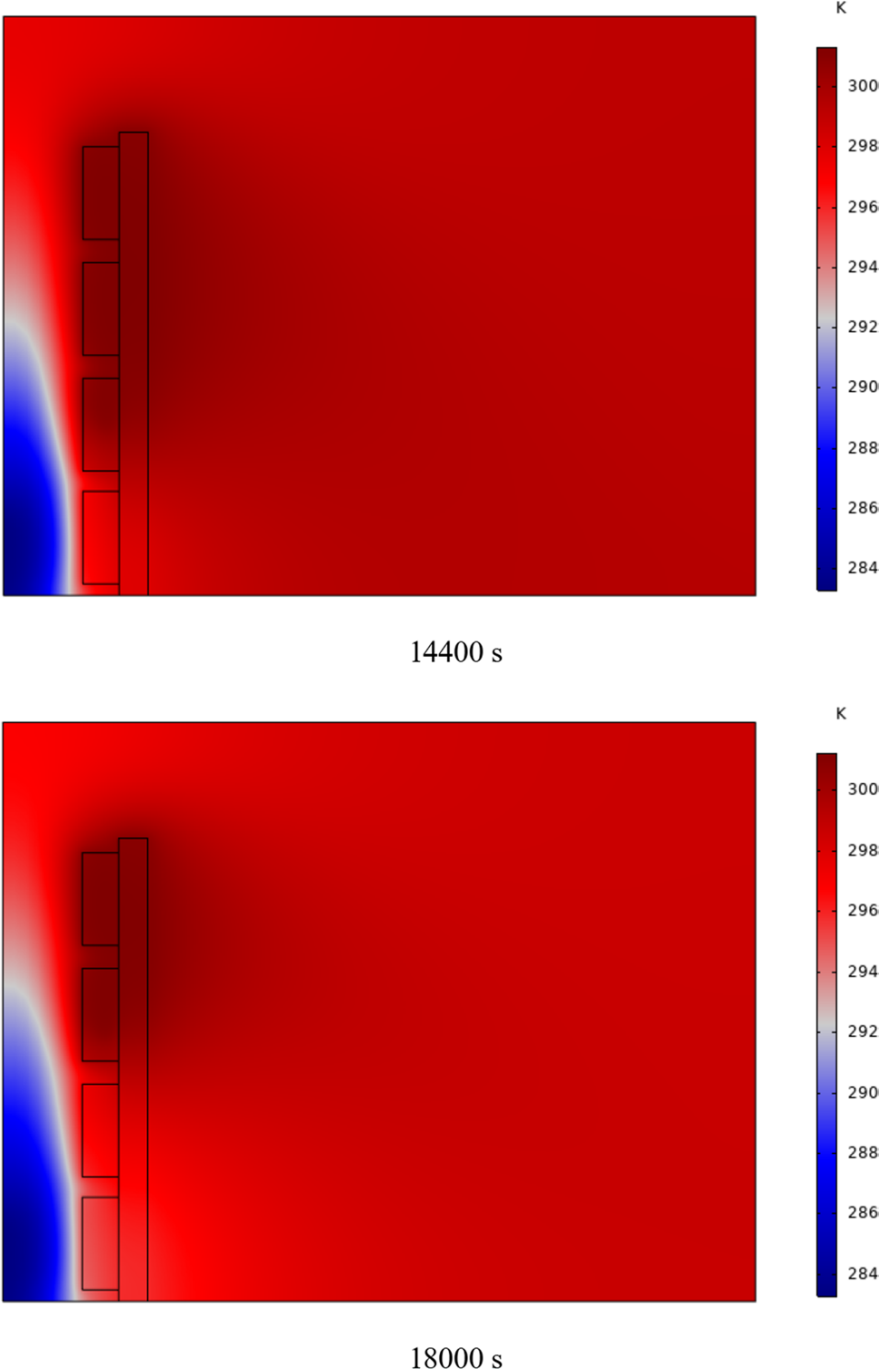

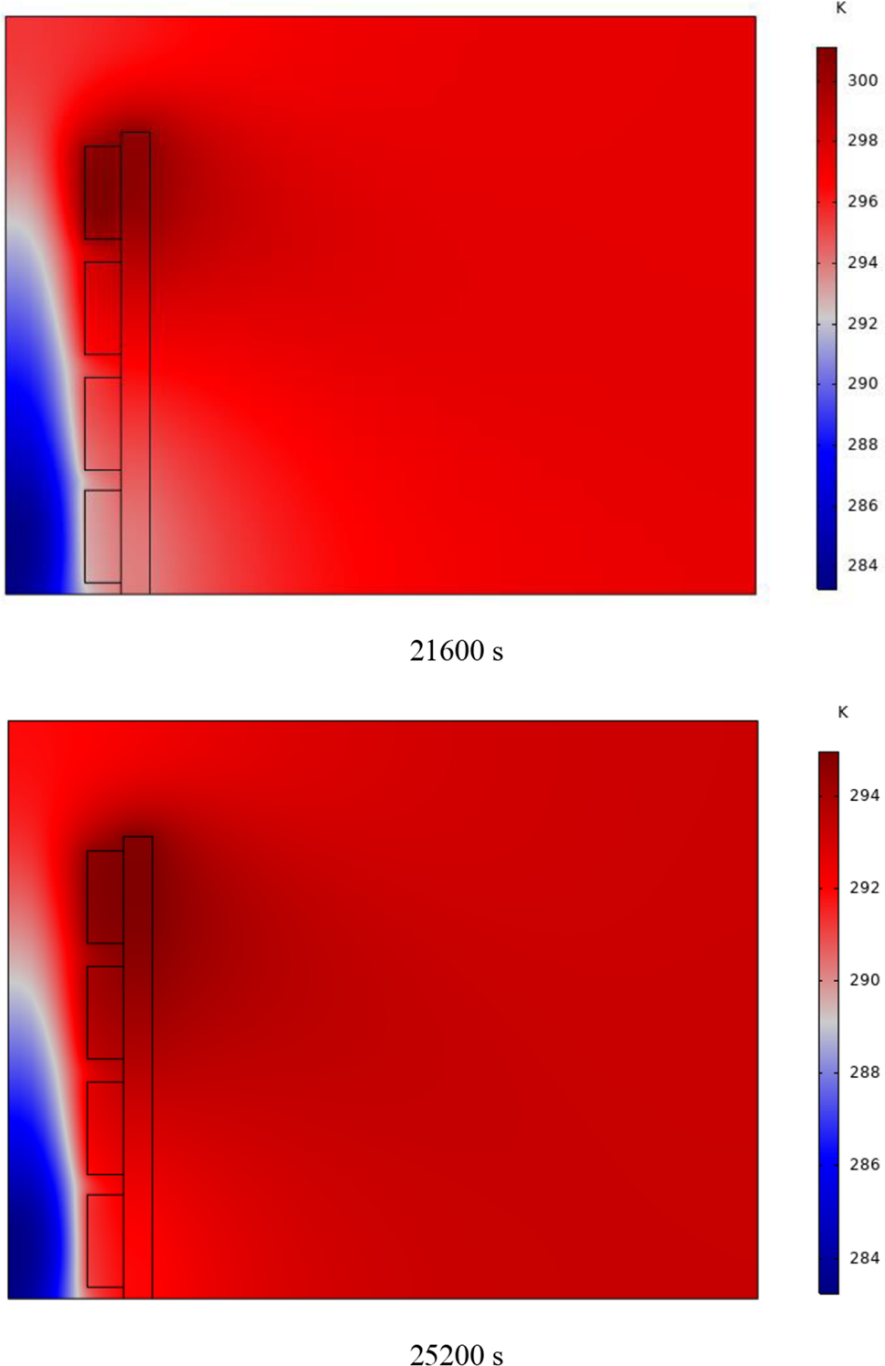
Temperature contour on the wall from 1 to 7 h for (D) 8 to 16 cm for RECs

The temperature contour on the wall is from 1 to 7 h for (D) 8 to 16 cm for RECs, as illustrated in Fig. 5. Cold air has experienced an increase in temperature after entering and hitting the wall and moving upwards. The latent energy inside the PCM is transferred to the air, and as a result, the air is heated, and the air that is warmer than the inlet is directed into the room. At the beginning of the process, due to the warmer wall and also more molten PCM in the obstacles, the TAR leaving the wall was higher. The temperature of the air leaving the wall falls with time and eventually approaches the temperature of the air entering the wall. The outlet air’s temperature is lower than the wall’s due to a decrease in the temperature of the wall and a reduction in the molten PCM in the cavities. As all PCM values solidify within the cavities, the TAR decreases sharply and closer to the inlet temperature.

**Fig. 6:**
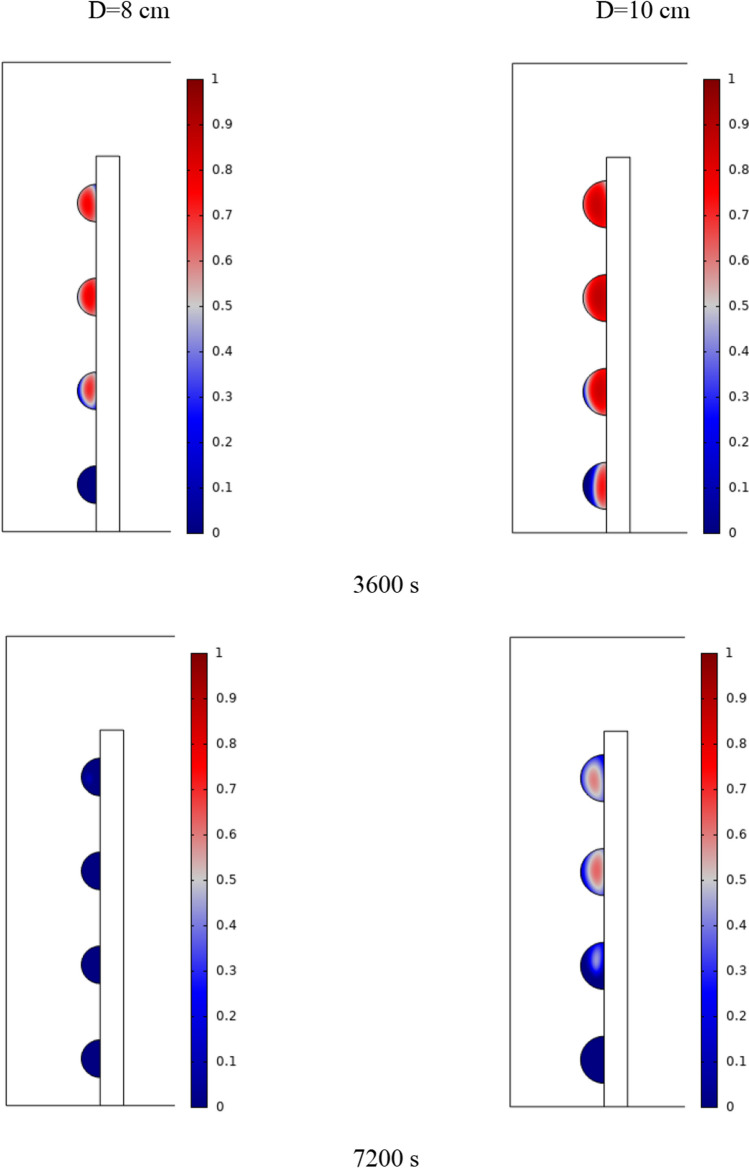

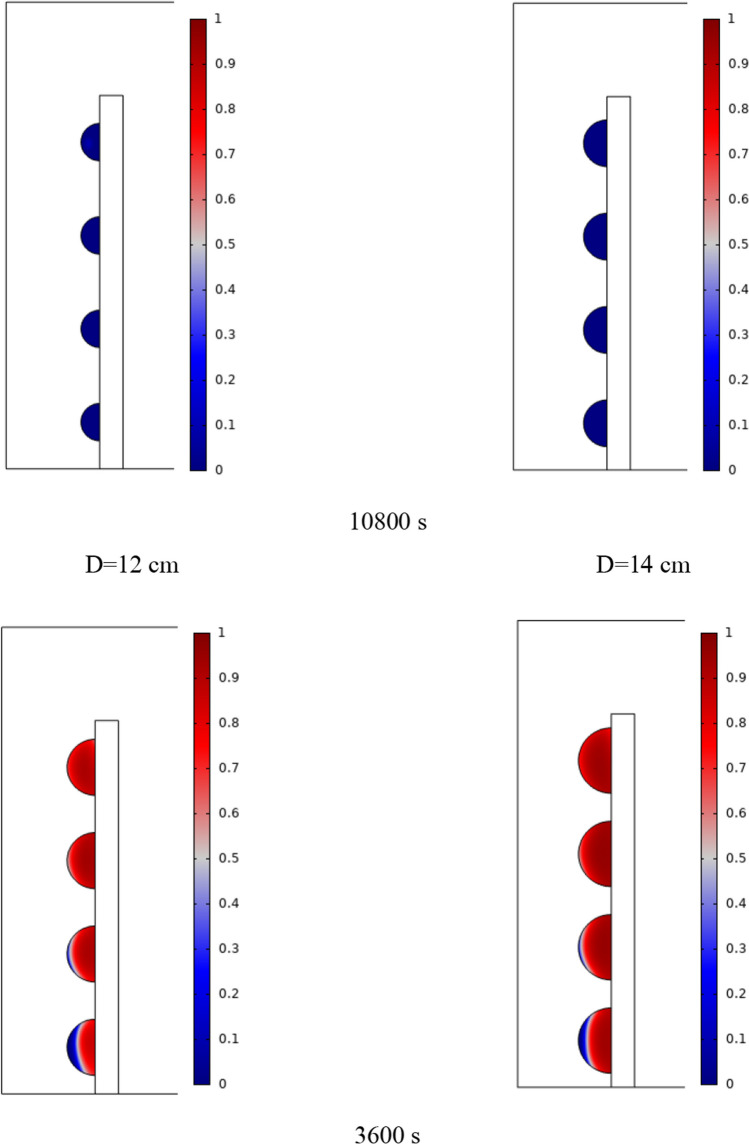

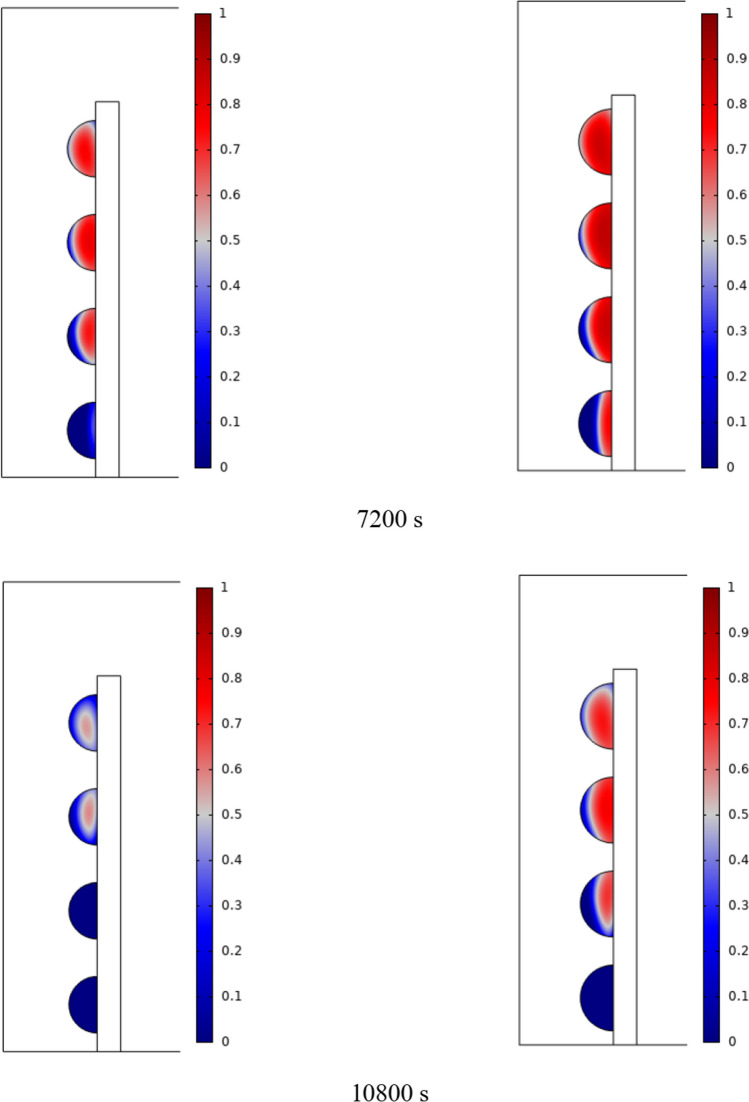

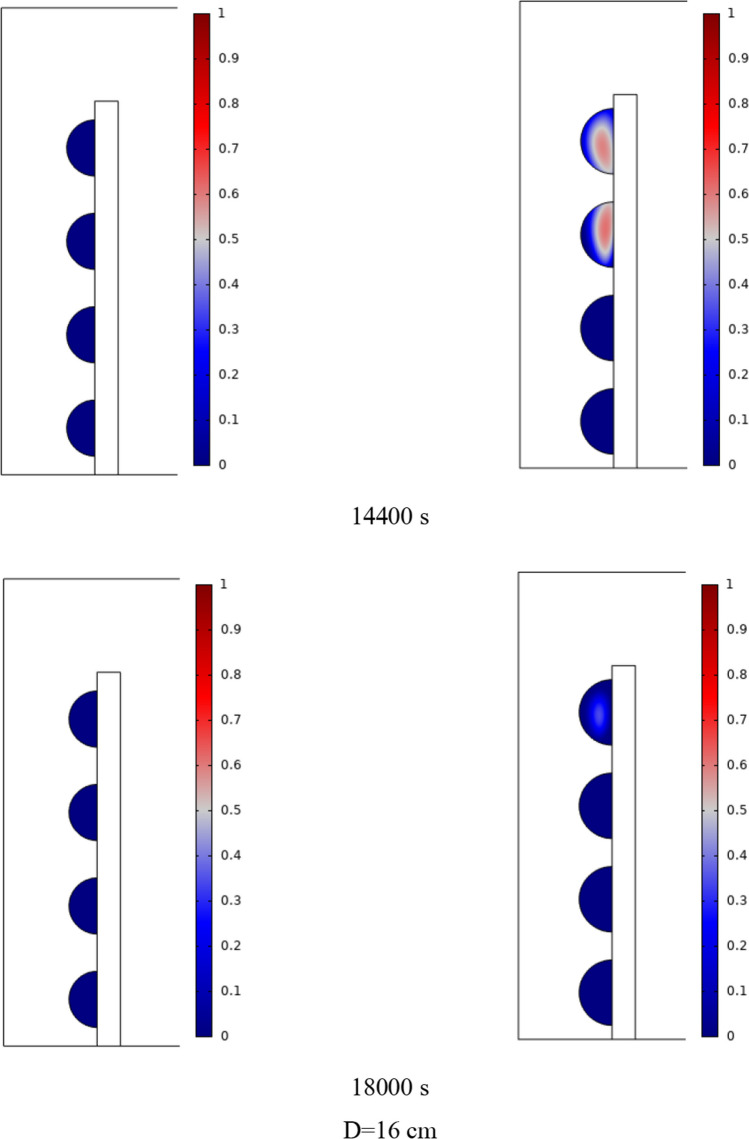

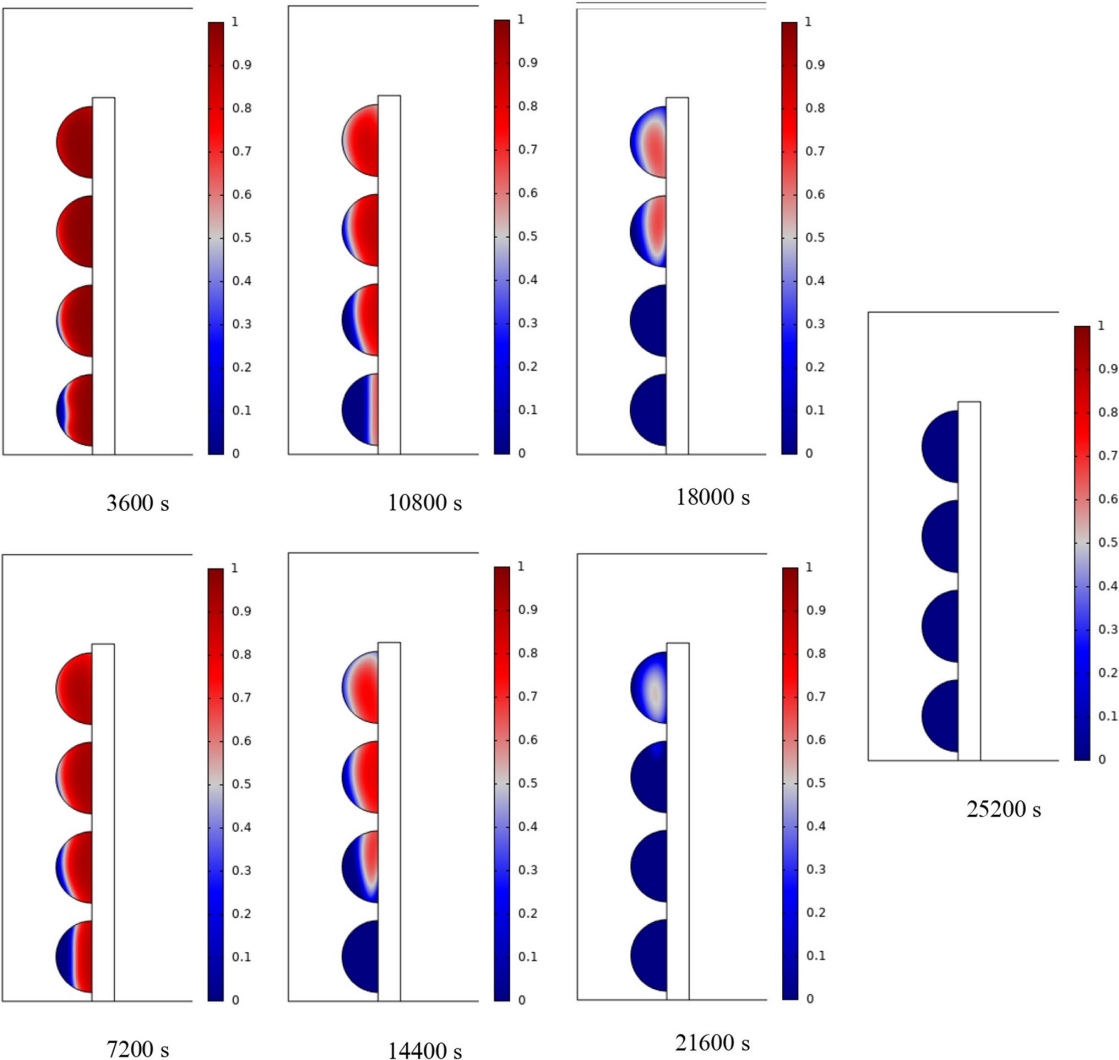
PCM freezing contour inside cavities, from different times, for D 8 to 16 cm for SECs

The PCM freezing contour within the cavities is shown in Fig. 6 for (D) 8 to 16 cm for SECs at various periods. A value of zero in the legend indicates a completely solid PCM, and a value of 1 indicates an entirely molten PCM. In SECs, the parts inside the SEC arc first lose their heat, and frozen PCM is seen. The lower barrier is initially frozen by air, and the upper barrier is solidified at the end. The larger the barrier, the more PCM there is in the barrier, and the longer it takes to solidify. The time required for all PCMs to solidify at an aspect ratio of 16 cm is much longer than the aspect ratio of 8 cm. In such a way that for an aspect ratio of 8 and 10 cm, less than 2 and 3 h were required for the complete freezing of PCM. The above two obstacles become completely solid in the last possible times.

**Fig. 7:**
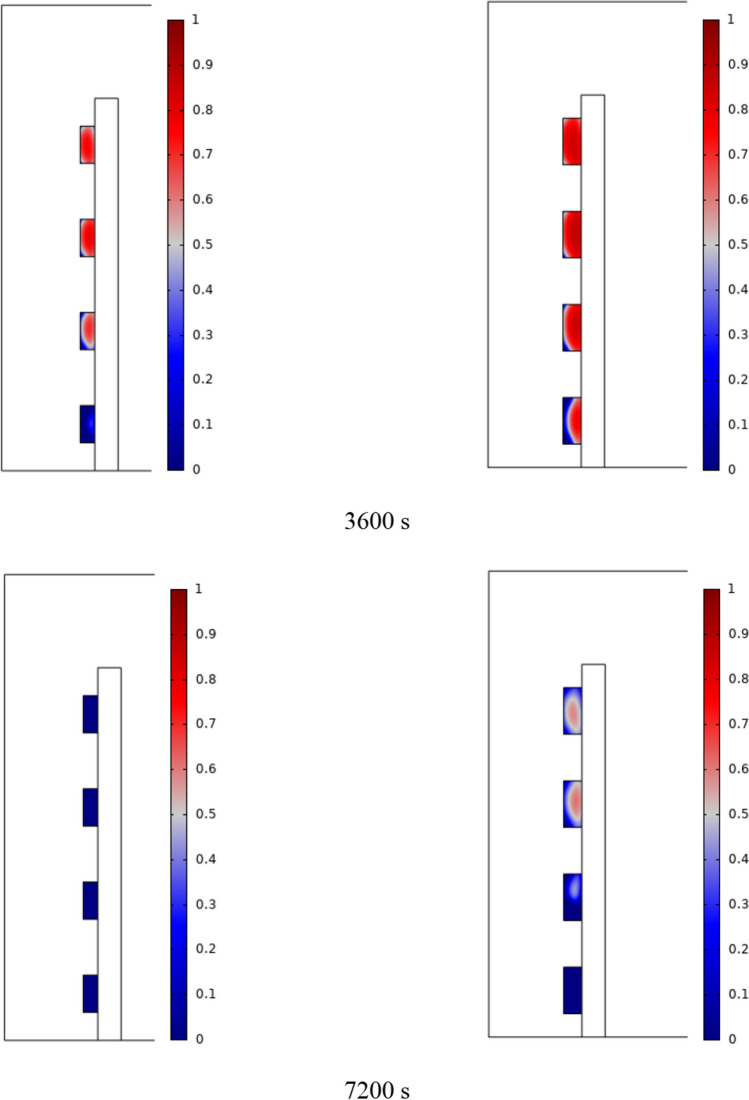

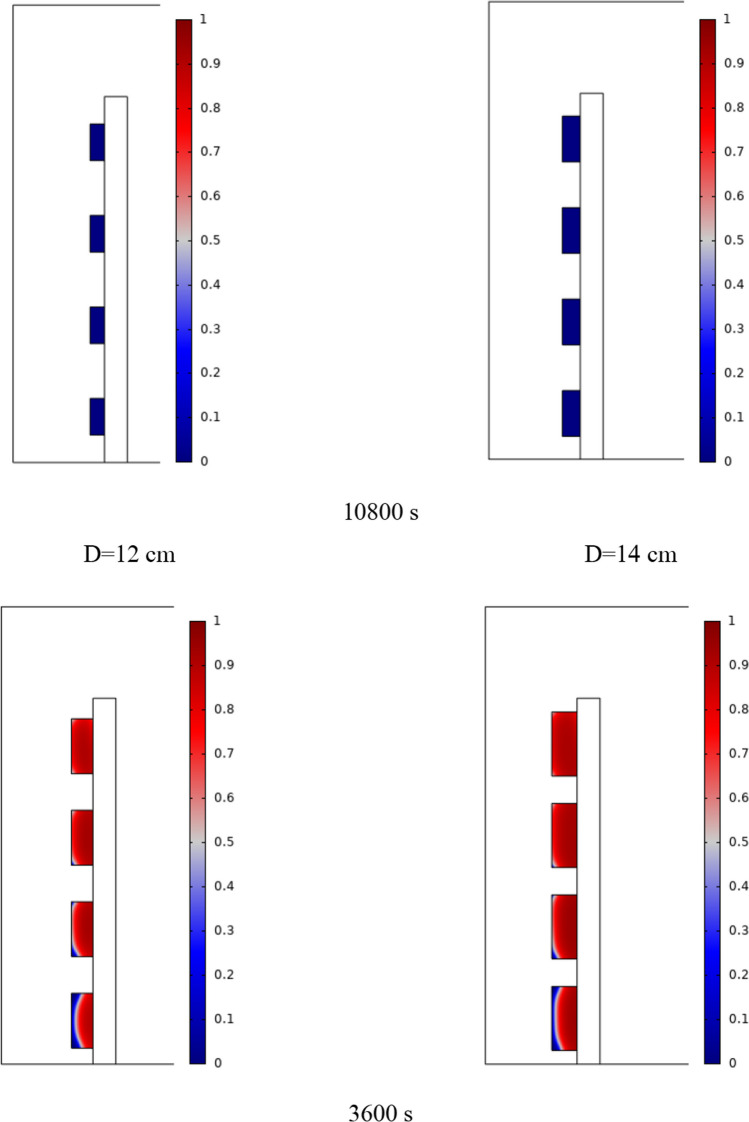

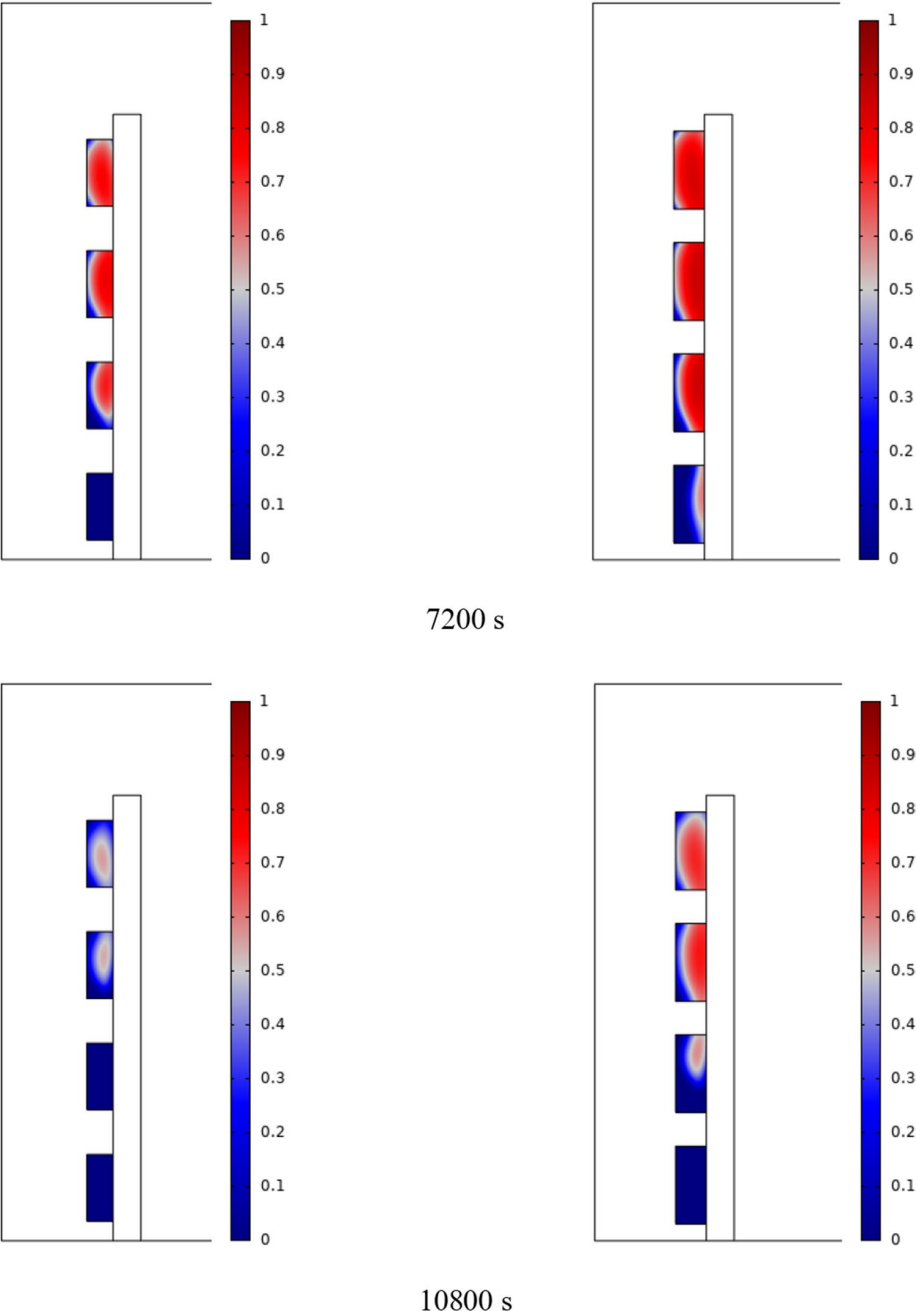

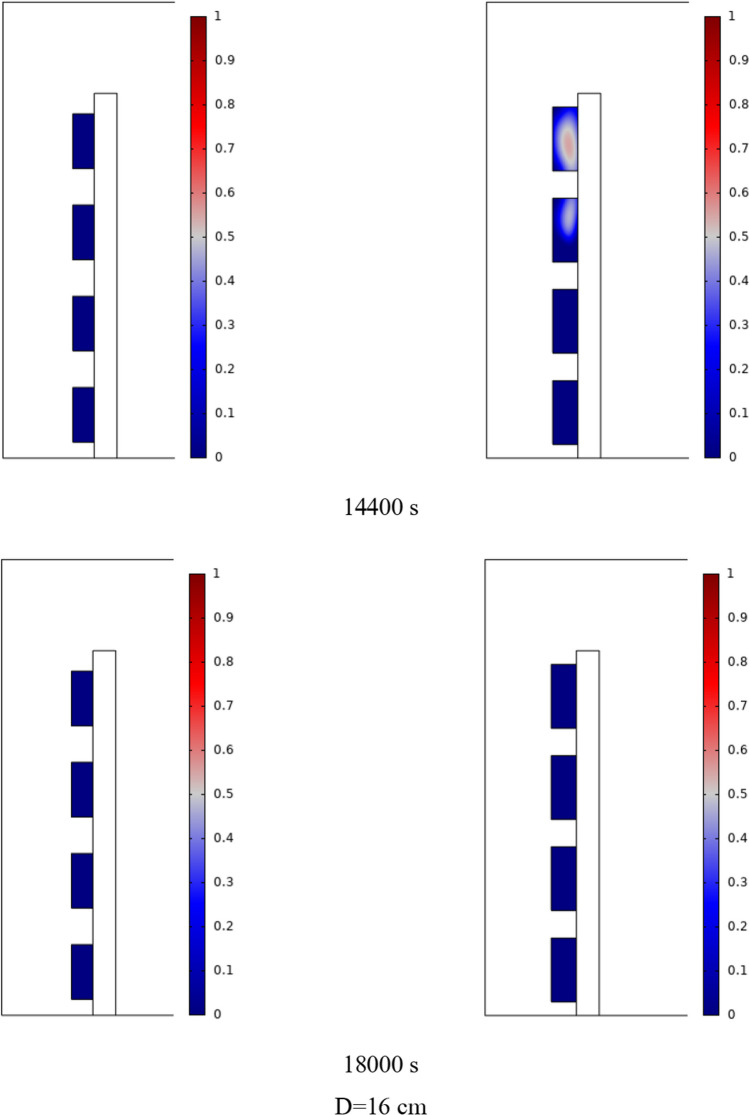

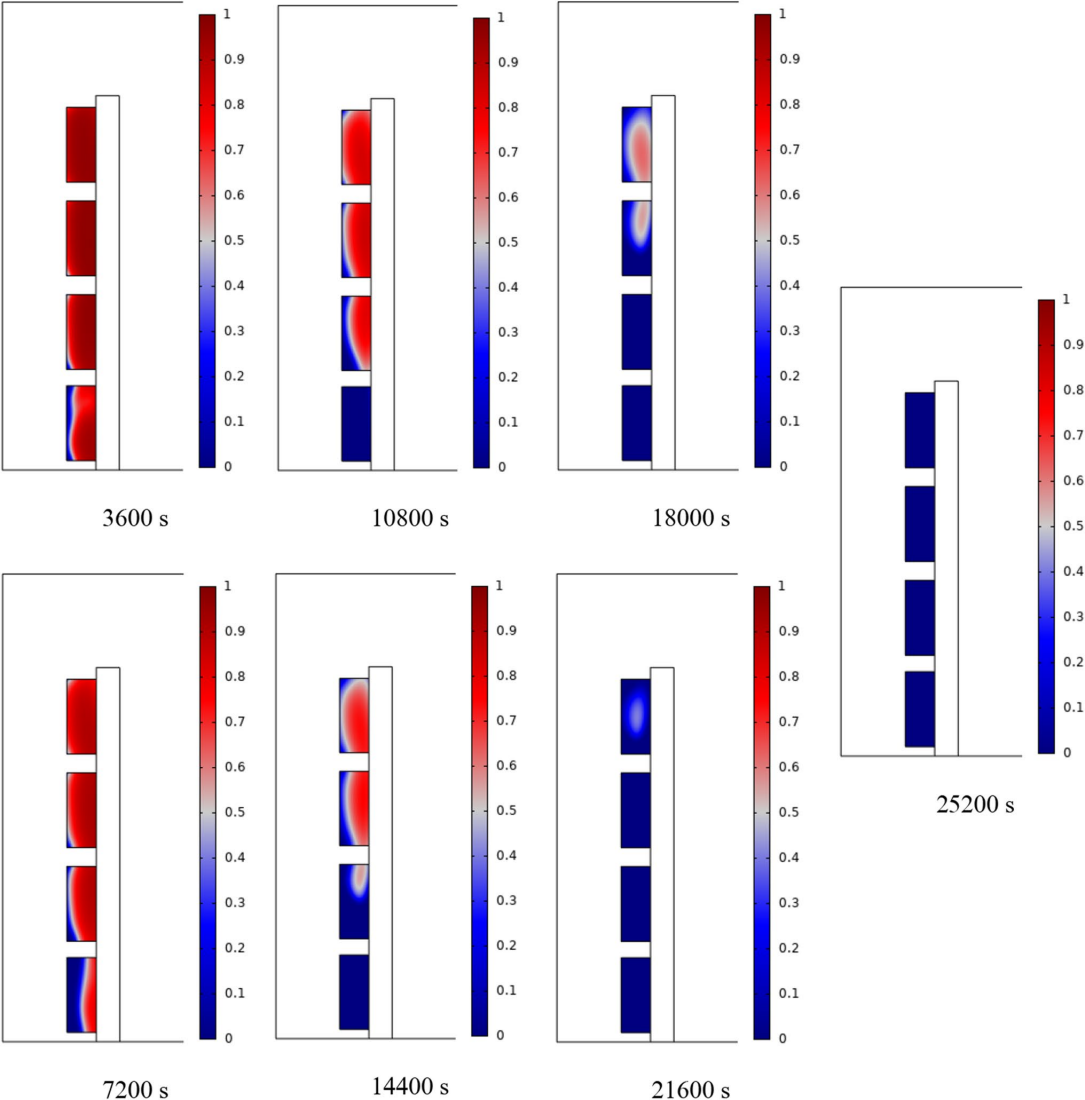
PCM freezing contour inside cavities, from different times, for (D) 8 to 16 cm for RECs

Figure 7 shows the PCM freezing contour inside the cavities, from different times, for (D) 8 to 16 cm for RECs. PCM solidification begins at the sharp corners of RECs and then gradually expands inward. The sharp corners of the cavities have more contact with the air, and as a result, they lose their heat faster and become a frozen PCM. The shortest time for PCM to freeze occurred at an aspect ratio of 8 cm, while the longest time for PCM to freeze occurred at an aspect ratio of 16 cm. Comparing the two shapes of RECs and SECs, it can be seen that the freezing time of PCM in RECs is a little less, and the freezing process shows a little faster in its RECs. Especially in the 16 cm aspect ratio in the freezing process, it was faster in REC than in SEC.

**Fig. 8:**
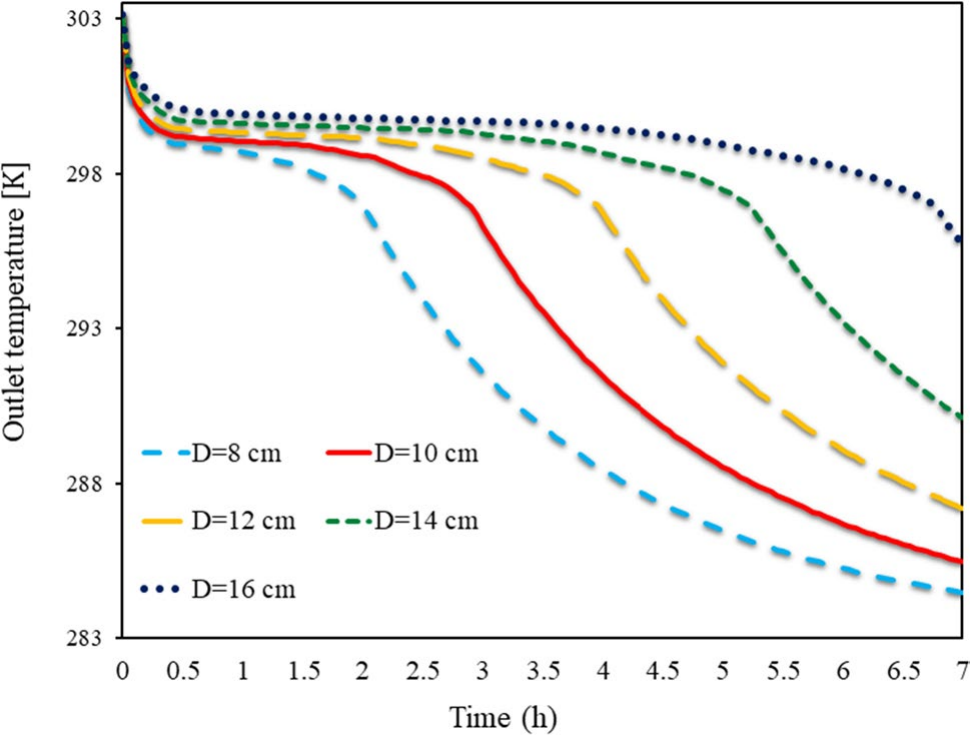
Exhaust TAR in front of the Trombe wall, in different(D)s from 8 to 16 cm, from 0 to 7 h for SECs

The output TAR from the front of the Trombe wall at various (D)s, from 8 to 16 cm, from 0 to 7 h, is shown in Fig. 8 for SECs. The TAR departing the wall is higher than the temperature entering the wall because of solar radiation. The energy stored in the wall and the PCM is trapped behind the cavities during the night when solar radiation fails to raise the TAR in front of the wall. As a result, the temperature of the exit air is higher than the temperature of the input air. The wall can only temporarily boost the TAR in front of it because of its limited heat capacity. The energy formerly stored in the PCM during the day could be released into the atmosphere at night, leaving the exhaust TAR over the wall. During the phase transition, the PCM holds onto a significant amount of energy in the form of latent heat of fusion. This energy is transferred to the air as latent heat over the night while turning molten PCM into solid PCM, increasing the TAR in front of the wall. In actuality, heat transfer from the PCM to the air takes longer the more PCM is used in the cavities.

**Fig. 9:**
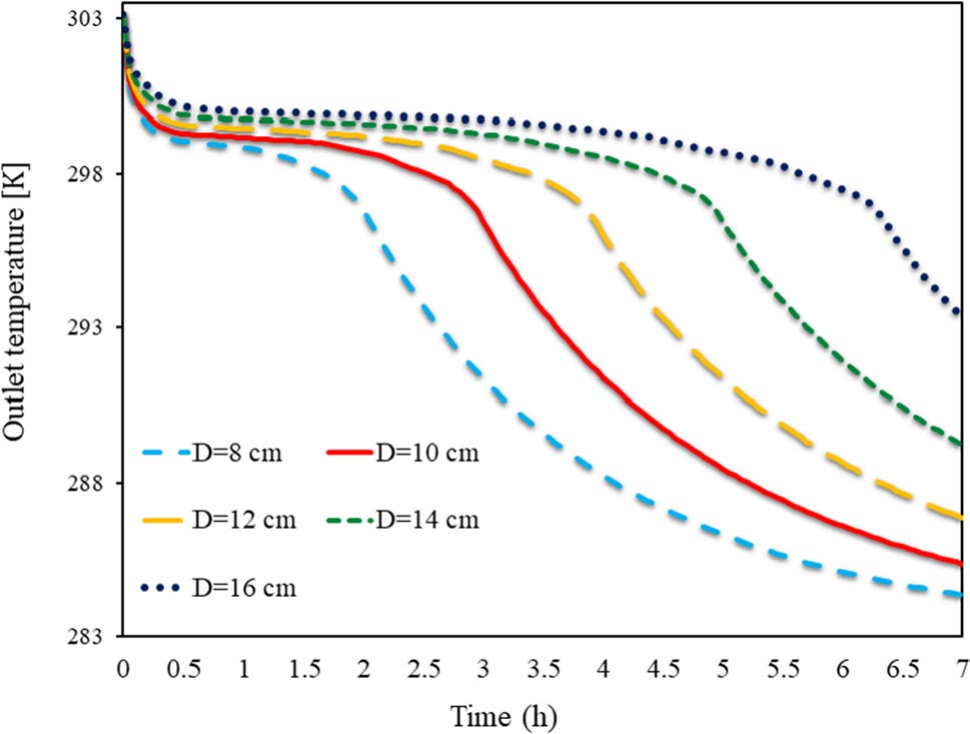
Exhaust TAR from the front of the Trombe wall in different (D)s from 8 to 16 cm from 0 to 7 h for RECs

The output TAR from the front of the Trombe wall at various (D)s from 8 to 16 cm from 0 to 7 h is shown in Fig. 9 for RECs. By creating airflow from the front of the wall, some of the latent energy in the PCM is transferred to the air, raising the TAR. Over time, some of the PCM on the barrier’s outside solidifies. When the PCM’s outer shell hardens, there is a little reduction in the heat transfer from the liquid PCM to the air. The amount of TAR has decreased with time yet the PCM cavities have not stopped melting. The latent energy in the wall is released when the amount of molten PCM in the cavities becomes zero, and the TWL rapidly decreases. During this time, the temperature of the wall’s exhaust air has been gradually dropping. More PCM being consumed in the wall allows the PCM to stay molten there for longer, which causes the exhaust air coming from the front of the wall to be hotter for longer. The TAR is greater at fixed times due to the employment of an SEC on the wall as opposed to a REC. As compared to the wall with RECs, the wall with SECs had a higher outlet TAR over a longer length of time. The usage of SECs has a more significant impact on the exit temperature of the wall, especially when the cavity dimensions are bigger.

**Fig. 10:**
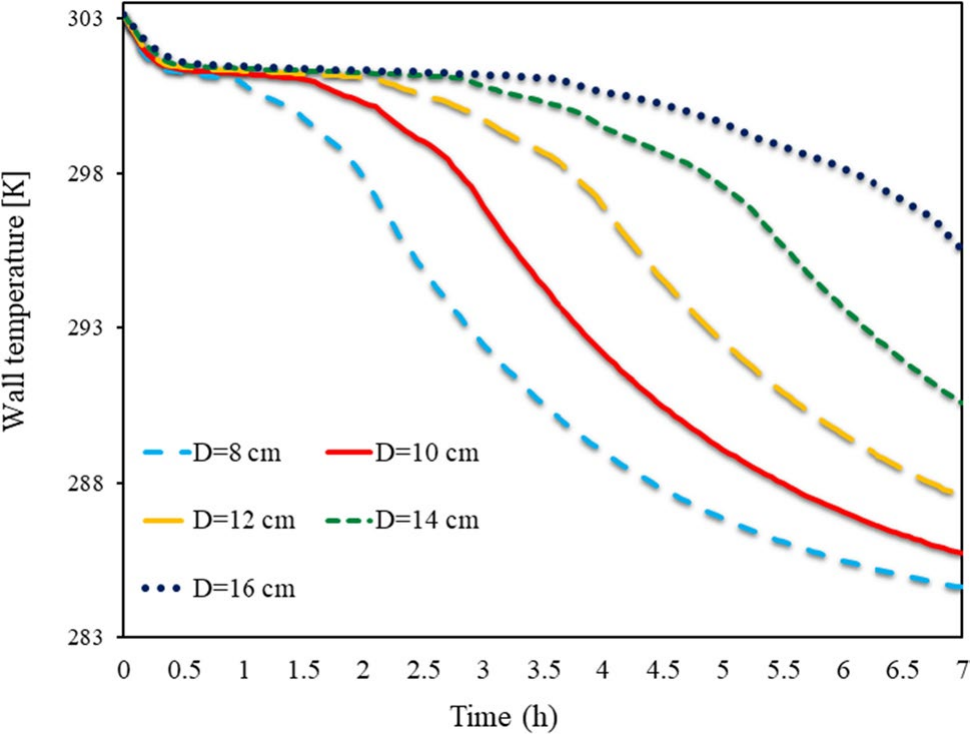
Trombe TWL in different (D)s, from 8 to 16 cm, from 0 to 7 h for SECs

As shown in Fig. 10, the Trombe TWL in different (D)s, from 8 to 16 cm, is shown from 0 to 7 h for SECs. This diagram shows the TWL along with the cavities. If a conventional wall was used in a short time, its temperature would decrease in the absence of solar, but due to the use of PCM on the wall, the TWL remained high for a longer time due to the volume of PCM used. The heat stored in the PCM due to the sun’s radiation during the night causes the TWL to remain at an acceptable value and can also heat the room. Using more PCM on the wall also saves more energy on the wall. In this way, the wall can maintain its temperature for a longer time. So, in the aspect ratio of 16, the TWL at all times has been higher than other aspect ratios of obstacles, so that in the aspect ratio of 16 cm, the temperature has been above 295.5 for 7 h.

**Fig. 11:**
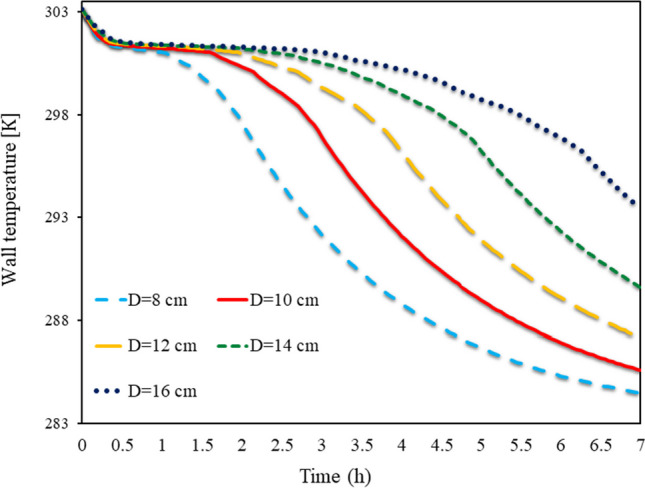
Trombe TWL in different (D)s from 8 to 16 cm from 0 to 7 h for RECs

Figure 11 depicts the Trombe TWL at different (D)s from 8 to 16 cm from 0 to 7 h for RECs. The TWL is highly dependent on the PCM temperature inside the cavities. It is this latent energy in PCMs that allows the wall to maintain its high temperature for a long time. Therefore, in walls with obstacles with a larger aspect ratio, both the TWL is higher, and the TWL remains high for a longer time. By comparing the placement of REC and circular obstacles on the wall, it can be seen that using SEC causes the TWL to remain high for a longer time. Also, the TWL at constant times for the wall with SECs is higher than the wall with RECs, so that in 7 h at a 16 cm aspect ratio, the use of SECs causes the TWL to reach 2.1 °C higher than the wall with RECs.

**Fig. 12:**
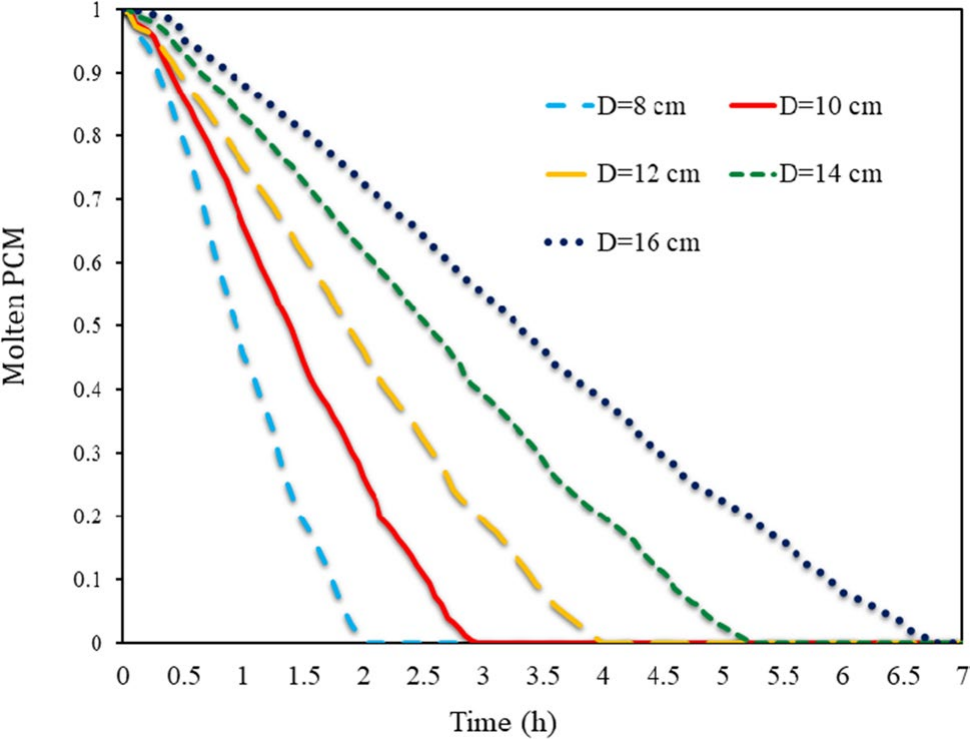
Volume fraction of molten PCM on average in all SECs at different (D)s from 8 to 16 cm from 0 to 7 h

In Fig. 12, in all SECs, the volume percentage of molten PCM is shown in various Ds ranging from 8 to 16 cm from 0 to 7 h. As long as thermal energy enters the PCM from outside the wall, the PCM remains molten. However, when the heat flux is cut off from outside the wall, the PCM freezing process begins due to the collision of cold air with obstacles. The process starts from the outer shell of the barrier and goes inside the barrier. Lower cavities freeze faster than upper cavities. By studying the average volume fraction of PCM, it can be seen that in smaller obstacles, due to the smaller volume of PCM, the whole PCM is frozen in less time. At constant periods, it can be shown that the quantity of molten PCM was greater when barriers with a bigger aspect ratio were employed. All PCMs in cavities with an 8 cm aspect ratio completely froze in a little under 2 h, but cavities with a 16 cm aspect ratio required more than 6.5 h to do the same.

**Fig. 13:**
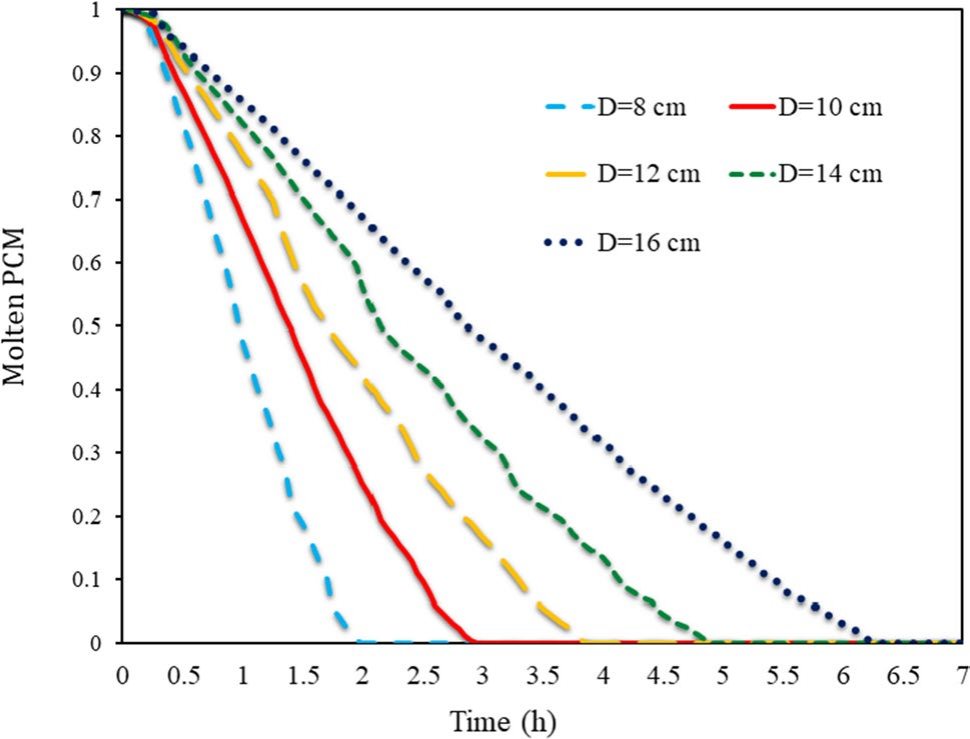
Volume fraction of molten PCM on average in all RECs in different (D)s from 8 to 16 cm from 0 to 7 h

In Fig. 13, the volume fraction of molten PCM is depicted on average in all RECs at different (D)s from 8 to 16 cm from 0 to 7 h. The air absorbs the latent energy inside the PCM, which causes the TAR to rise and the PCM to solidify inside the cavities. Smaller cavities on the wall have less PCM and therefore freeze entirely in less time. However, larger cavities require more time for the entire PCM to solidify. Comparing the two RECs and SECs, it can be seen that the time required for PCM solidification within cavities was longer for SECs than for RECs. Especially at higher aspect ratios, the increase in PCM freezing time was more dramatic. The time required for PCM solidification at a 16 cm aspect ratio in semi-circular obstacles was 15 min longer than in RECs.

## Conclusion

In this paper, the effect of using two types of SEC and RECs filled with PCM on a Trombe wall on the TWL, the complete freezing time of PCM, and the TAR leaving the wall have been numerically analyzed. Also, TWL contours and PCM solidification front progress in cavities have been studied. The outcomes of this investigation, which was conducted for 5 various cavity dimensions, are as follows:

1. In general, the time required for PCM solidification in SECs is more than in RECs. However, in most cases, the time required to freeze the PCM at a 16 cm aspect ratio was 15 min longer in SECs than in RECs.
2. The TWL at constant times for the wall with SECs is higher than the wall with RECs, so that in 7 h at a 16 cm aspect ratio, the use of SECs causes the TWL to be 2.1 °C higher than the wall with RECs.
3. In the aspect ratio of 16, the TWL was higher than the other aspect ratios of the obstacles at all times, so that in the SEC, in the aspect ratio of 16 cm, after 7 h, the temperature was above 295.5.
4. For a longer time than the wall with RECs, the wall with SECs had a higher TAR. Especially in larger dimensions of cavities, the use of SECs has a better effect on the exit temperature of the wall.

## Data Availability

Not applicable.
